# Orbital contributions to magnetically induced current densities using gauge-including atomic orbitals†

**DOI:** 10.1039/d5sc00627a

**Published:** 2025-04-01

**Authors:** Rinat T. Nasibullin, Maria Dimitrova, Rashid R. Valiev, Dage Sundholm

**Affiliations:** a Department of Chemistry, Faculty of Science, University of Helsinki P. O. Box 55 (A. I. Virtanens plats 1) FIN-00014 Finland Dage.Sundholm@helsinki.fi Rinat.Nasibullin@helsinki.fi

## Abstract

We have developed a method to calculate orbital contributions to magnetically induced current density (MICD) susceptibilities in molecules using gauge-including atomic orbitals (GIAO). The methods implemented in the GIMIC program have been used for analyzing orbital contributions to magnetically induced ring-current (MIRC) strengths. We have studied five aromatic, one nonaromatic, and four antiaromatic molecules. We show here that the contributions to the MIRC strength of all orbitals belonging to a given irreducible representation of the molecular point group in the presence of an external magnetic field are divergence free, whereas the MICD susceptibility of the individual orbitals are generally not divergence free. The largest contribution to the MIRC strength of antiaromatic molecules originates from the transition between the highest occupied molecular orbital (HOMO) and the lowest unoccupied molecular orbital (LUMO), whereas aromatic molecules have significant contributions involving many occupied orbitals. The MIRC contributions of σ orbitals are significant for planar molecules with a strained molecular structure.

## Abstract 2

We have developed a method to calculate orbital contributions to magnetically induced current density susceptibilities using gauge-including atomic orbitals.

## Graphical abstract

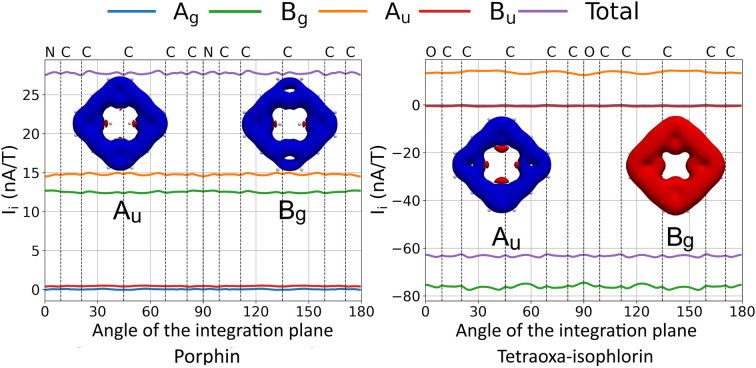

## 1 Introduction

The aromaticity concept was originally introduced to characterize molecules whose electron-delocalization properties resemble those of benzene.^1,2^ The electron delocalization of aromatic molecules leads to enhanced thermodynamic stability, reduced bond-length alternation, particular reactivity, and typical magnetic and spectroscopic properties.^3^ The aromatic nature according to the magnetic criterion can be determined by measuring or calculating proton nuclear magnetic resonance (^1^H NMR) chemical shifts or by calculating various magnetic response properties such as nucleus-independent chemical shifts (NICS),^4^ magnetically induced current density (MICD) susceptibilities,^5–11^ and magnetically induced ring currents (MIRC).^11–15^

These computational approaches have to some extent been used for estimating contributions to the aromatic nature of individual molecular orbitals.^16–23^ A general and reliable method for calculating orbital contributions to MICD susceptibilities and molecular properties would be useful, since decomposition into orbital contributions provides insights into the electronic structure and can be used for predicting changes due to substitution, structural variations, or charge redistribution. Common approaches to qualitatively understand the role of the frontier molecular orbitals (MO) in aromatic bonds comprise analyses of orbital energies and spatial distributions of orbitals as well as using symmetry selection rules.^24,25^

Symmetry-based selection rules of the transitions between occupied and virtual orbitals have been used for assessing which occupied orbitals contribute to the MICD susceptibilities and magnetic properties. When the electronic transition between the highest-occupied molecular orbital (HOMO) and the lowest-unoccupied molecular orbital (LUMO) is electric-dipole (translationally) allowed, ring-shaped molecules may sustain a diatropic MIRC in the presence of an external magnetic field. Aromatic molecules can be identified using group theory. Molecules can be aromatic when the product of the irreducible representations of the HOMO, the LUMO and the translation operator contains the total symmetric irreducible representation. Analogously, molecules can be antiaromatic when the irreducible representation of the rotational operator multiplied by the irreducible representations of the HOMO and LUMO contains the total symmetric irreducible representation. The transition is then magnetically dipole (rotationally) allowed, which eventually leads to a paratropic MIRC.^26–28^ Nonaromatic molecules are in this context conjugated molecular ring that are neither aromatic nor antiaromatic. Even though a transition is formally translation or rotationally allowed, a molecule may still be nonaromatic due to a large spatial separation of the occupied and virtual orbitals or because the energy separation between them is large.

The symmetry analysis provides a qualitative interpretation of molecular aromatic properties, whereas the MIRC strengths provide quantitative information about the aromatic nature because molecules sustaining a net diatropic MIRC are aromatic, whereas antiaromatic molecules have a net paratropic MIRC.^29^ The MIRC strength can be used for determining how strongly aromatic or antiaromatic the molecule is.^11^

Orbital contributions to the MICD susceptibility have previously been calculated using the continuous transformation of the origin of the current density (ipsocentric) approach.^7,10,30^ In the ipsocentric approach, the origin of the vector potential of the external magnetic field is the position where the MICD susceptibility is calculated. Steiner and Fowler performed the first calculations of orbital contributions to the MICD susceptibility using the few-orbital model.^24,31^ They studied the MICD susceptibility of benzene with the ipsocentric approach using an early version of the SYSMOIC package.^14^ The ipsocentric approach employs atomic basis functions that do not depend on the magnetic perturbation, whereas gauge-including atomic orbitals (GIAO) also known as London atomic orbitals incorporate the gauge origin in the basis functions^32,33^ leading to a fast basis-set convergence of the MICD susceptibility and NMR shielding constants.^34,35^

The gauge-including magnetically induced currents (GIMIC) method employs GIAOs.^11–13,15,28^ GIMIC calculations need the one-electron density matrix and the three magnetically perturbed density matrices as input. The density matrices can be calculated at various levels of theory using common quantum chemistry program packages. Cartesian coordinates of the molecular structure and basis-set information are also needed as input data. The GIMIC method has been successfully applied in MICD susceptibility studies of aromatic and antiaromatic molecules including planar molecular rings,^29^ Möbius-twisted molecular rings,^36,37^ toroidal molecules,^38^ organometallic molecules,^39–41^ and inorganic molecular clusters.^42,43^

MICD susceptibilities calculated with GIMIC suffer from a tiny charge leakage, *i.e.*, the divergence of the MICD susceptibility does not vanish when using finite basis sets. Divergence-free MIRC strengths can be calculated by performing numerical line integration of the induced magnetic field along the stagnation line of the MIRC vortex using the Ampère-Maxwell law.^44^ However, it has been shown that the charge-leakage error of GIMIC calculations is negligibly small already when using commonly used moderate-size basis sets and that it practically vanishes when using large basis sets.^11^ Recently, an expression for correcting the divergence was derived enabling calculations of divergence-free MICD susceptibilities using any basis set.^45^

NMR shielding tensors have been partitioned into contributions of various orbitals by performing natural chemical shielding (NCS) analyses.^46^ However, unitary transformations of the orbitals lead to spurious magnetic shielding contributions,^25^ which can be identified as unphysical core and valence-σ contributions to the MICD susceptibility and to the NMR shielding tensors due to the mixing of occupied orbitals belonging to the same irreducible representation.^43,47^ Orbital contributions to the MIRC strengths can also be estimated by partitioning the MICD susceptibility.^25,48^ Orbital contributions and atomic orbital contributions to NMR shielding tensors can then be calculated by numerical integration.^49,50^

In this work, we report an extension of the GIMIC method that enables calculations of orbital contributions to the MICD susceptibility. This is achieved by transforming the density matrices in the basis-function (atomic) basis to the canonical orbital basis where molecular orbital contributions can be identified. The selected orbital contributions to the density matrices are transformed back to the basis-function basis. The orbital contributions to the MICD susceptibility can be analyzed and visualized in the same way as the total MICD susceptibility.

We have studied eleven planar molecules and one nearly planar monocyclic molecule with different types of aromatic character. Six of them are aromatic (benzene (*D*_6h_), borazine (*D*_3h_), 1,5-dibora-2,4-diazabenzene (*C*_2v_), porphin (*D*_2h_), cyclopropane (*D*_3h_) and the cyclopropenium cation (*D*_3h_)), one is nonaromatic (1,4-cyclohexadiene, (*D*_2h_)), and four are antiaromatic (cyclooctatetraene (*D*_4h_ and *D*_2d_), hexadehydro[12]annulene (*D*_3h_), tetraoxa-isophlorin (*D*_2h_) and cyclobutadiene (*D*_2h_)), where the molecular point group is given within parenthesis.

Cyclobutadiene and the cyclopropenium cation were studied because they have bond angles that significantly deviate from the standard bond angles of sp^2^ hybridized hydrocarbons leading to ring strains. These strained molecules, along with molecules like cyclopropane, cyclobutane, and cubane, sustain an MIRC in the valence σ orbitals leading to what is called σ aromaticity or σ antiaromaticity. The direction (tropicity) of the MIRC is determined by the number of electrons in the σ orbitals of the C–C bonds.^29,51–59^

The results for some of the molecules are compared to available data calculated using the ipsocentric approach. Orbital contributions to the MICD susceptibility of most of the studied molecules are reported here for the first time to the best of our knowledge.

The method we use for calculating orbital contributions to the MICD susceptibility is described in Section 2. We describe the employed computational levels in the Section 3. The obtained results are presented in Section 4. The main results are summarized in Section 5, which also contains the most important conclusions of our study.

## 2 Methodology

### 2.1 Decomposition of density matrices

The one-electron density matrix in the atomic-orbital basis (**D**^AO^) consist of contributions of the individual occupied orbitals1
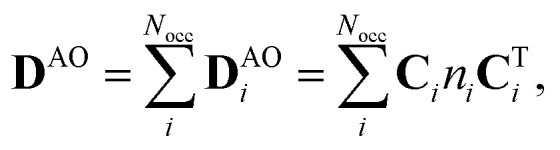
where *N*_occ_ is the number of occupied orbitals and *n*_*i*_ is the occupation number of MO *i*, and **C**_*i*_ contains the coefficients of MO *i*. The orbital contributions to **D**^AO^ (**D**^AO^_*i*_) can be identified by transforming it to the canonical-orbital basis **D**^MO^. The basis transformation is done by multiplying from right with **SC**, where **S** is the overlap matrix and **C** is matrix containing the MO coefficients, and with its transpose **C**^T^**S** from left

2 **D**^MO^ = **C**^T^**SD**^AO^**SC**.

The first-order magnetically perturbed density matrices in the AO basis for each Cartesian direction of the magnetic field (*B*_*λ*_, *λ* ∈ {*x*, *y*, *z*}) can be obtained from the MO coefficients and the first-order change of the MO coefficients (**C**_*λ*_) due to the external perturbation *B*_*λ*_3
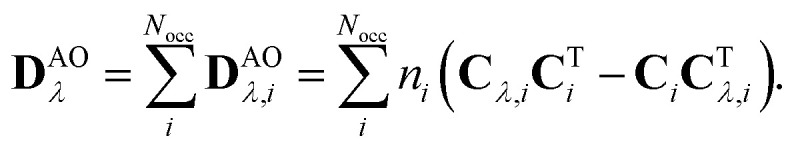
**C**_*λ*_ can be expressed as a unitary (**U**_*λ*_) transformation of the unperturbed occupied MO coefficients4
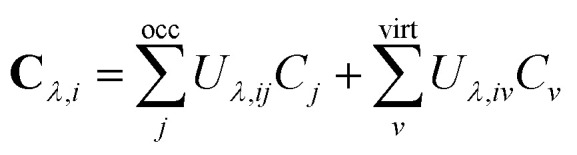
which is the orbital response due to the external magnetic field *B*_*λ*_ in the limit of vanishing perturbation.^60–62^ The occupied–virtual block of **U**_*λ*_ (*U*_*λ*,*iv*_) is obtained by solving the orbital response equations. The virtual–virtual block of **U**_*λ*_ does not contribute to the perturbed density matrices.^63^ The occupied–occupied block of **U**_*λ*_ (*U*_*λ*,*ij*_) does not vanish when perturbation-dependent GIAOs are used.^64^ However, it can be determined *i.e.*, by differentiating the orthonormalization condition^60,61^5
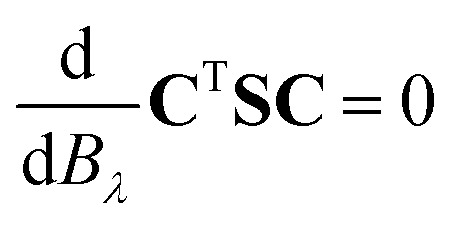


Using the relation in eqn (4), the final expression for the unitary transformation matrix can be written as^65^6
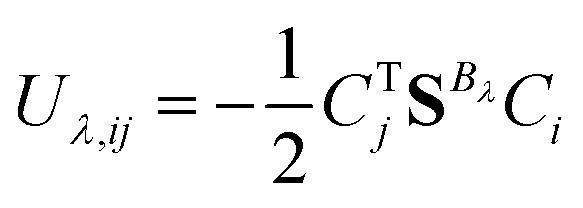
where **S**^*B*_*λ*_^ is the first derivative of the overlap matrix with respect to the external magnetic field (*B*_*λ*_) in the limit of vanishing perturbation. The contributions of orbital *i* to the perturbed density matrices can also be obtained by performing the same unitary transformation and orbital selection procedure as applied on the one-electron density matrix. The orbital contribution to the perturbed density matrices in the MO basis consists of one row and one column of the matrices. The common elements of two orbitals in the occupied–occupied block are shared equally between the two orbitals.

The orbital contributions to the density matrices are transformed to the AO basis. The dimension of the AO density matrices is *N*_basis_^2^, where *N*_basis_ is the number of basis functions. The orbital contributions to the MICD susceptibility can then be calculated with GIMIC in the same way as the total MICD susceptibility by using the corresponding orbital contributions to the density matrices **D**^AO^_*λ*,*i*_ and **D**^AO^_*i*_ as input. Contributions to the MIRC strength of the *i*^th^ MO are obtained by modifying the occupation matrix *n*_*i*_ and performing the linear transformations and thereby avoiding time-consuming calculations.

The computational costs for calculating orbital contributions to the MICD susceptibility are comparable to those of all-electron GIMIC calculations because the computational time for preparing the density matrices in the AO basis for selected orbitals is much smaller than for the GIMIC calculation, whose computational time is independent of whether it is the total MICD susceptibility or orbital contributions to it.

The perturbed density matrices contain information about the response of all orbitals through the mixing of occupied and virtual orbitals, whereas in calculations of orbital contributions using the few-orbital model, only selected virtual orbitals are considered.^24,25,31^ The selection might lead to wrong conclusions because many small contributions from pairs of occupied and virtual orbitals may affect the interpretation. We can perform calculations using the few-orbital model by identifying the orbital response involving selected occupied and virtual orbitals, whereas the rest of the response terms are set to zero. We have not implemented the approximate few-orbital model because the computational costs are the same as when considering the response of all orbitals.

### 2.2 Group theoretical analysis

The external magnetic field reduces the symmetry of the molecular point group when symmetry operations of the molecular point group do not map the magnetic field onto itself.^66,67^ The resulting molecular point group in the presence of the external magnetic field is a subgroup of the molecular point group in the absence of the field and of *C*_∞h_,^68^ which is the point group of the external magnetic field. The order of the subgroup is usually larger than one, when the magnetic field coincides with a symmetry axis of the molecular point group.^66^ The point group of the molecular structure in the presence of an external magnetic field can conveniently be determined by using the flow chart reported in ref. 66.

Orbital contributions to the MICD susceptibility are not in general divergence free, whereas the orbital contributions of all orbitals in a given irreducible representation of the combined point group are divergence free. Thus, the sum of the orbital contributions to the MIRC of all orbitals in each irreducible representation is then charge conserving, which leads to an MIRC whose strength is independent of the orientation of the integration plane.

The point groups of the studied molecules in the presence of an external magnetic field along the main symmetry axis, which is perpendicular to the molecular ring, are *C*_6h_ (benzene), *C*_4h_ (planar cyclooctatetraene), *S*_4_ (bent cyclooctatetraene), *C*_3h_ (borazine, cyclopropenium cation, and hexadehydro[12]annulene) , *C*_2h_ (porphin, 1,4-cyclohexadiene, tetraoxa-isophlorin and cyclobutadiene), and *C*_s_ (1,5-dibora-2,4-diazabenzene).

### 2.3 Spatial averaging of the orbital contributions

The MICD (**J**(**r**)) is obtained by contracting the MICD susceptibility tensor with the direction vector of the external magnetic field. For a given direction of the external magnetic field, the circulation direction (tropicity) of the MIRC vortex can be in the classical diatropic (aromatic) direction, which is here the positive direction or in the negative paratropic (antiaromatic) direction. The tropicity can be assessed by visualizing the vector field. The profile of the MIRC and its strength (*I*) can be obtained by numerically integrating **J**(**r**) that passes through a surface (**S**) cutting the molecular ring.7
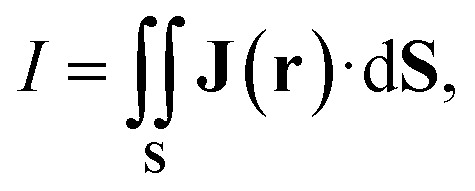
where the dot represents the scalar product of **J**(**r**) with the normal of the infinitesimal cross-section area d**S**. The two-dimensional integration domain begins in the center of the MIRC vortex and extends to a sufficiently large distance from the molecule in the other spatial directions. Some inaccuracy is introduced when the stagnation line of the MIRC vortex and the edge of the integration plane do not coincide. However, the studied molecules have high symmetry implying that the magnetic field, the stagnation line of the MIRC vortex and the main symmetry axis coincide. The integration plane is usually positioned so that it cuts the ring in the middle of a chemical bond. However, since charge conservation is almost fulfilled when using GIAOs and standard basis-set sizes, *I* is only weakly dependent on the position of **S**.^69^ The tropicity of **J**(**r**) can be identified and separated into diatropic and paratropic contributions by following the vector field around the vortices of **J**(**r**) using *e.g.*, the Runge–Kutta algorithm.^70^

**Fig. 1:**
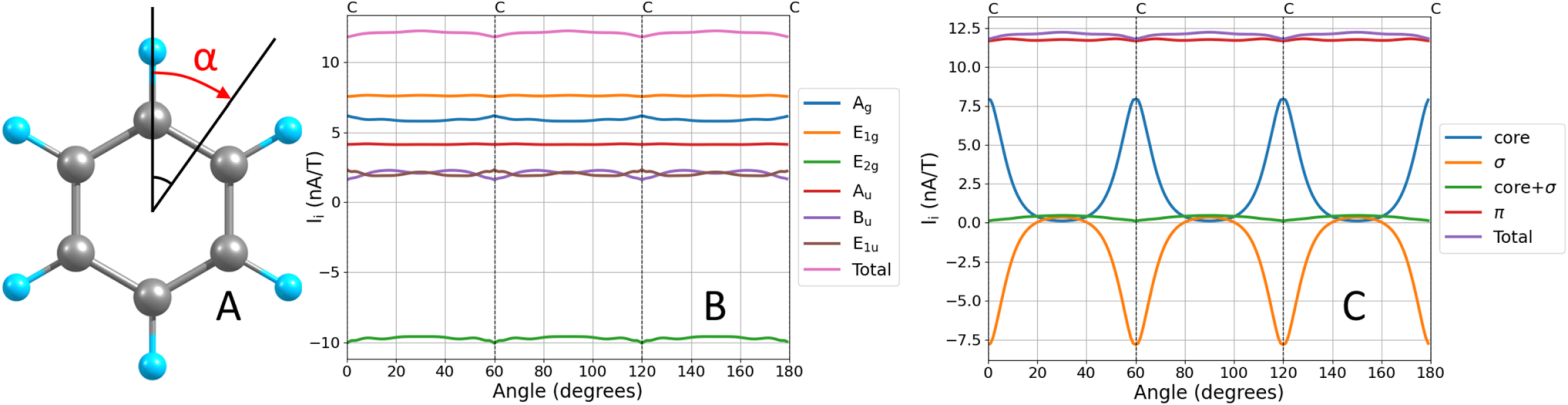
The orientations of the integration plane for calculating the angular dependence of the MIRC strength of benzene (A). The angular dependence of the MIRC strength of benzene for all orbitals in each irreducible representation of the *C*_6h_ point group (B). The angular dependence of the MIRC strength of the core, valence σ, and π orbitals of benzene (C).

Since contributions to the MIRC strength of individual orbitals (*I*_*i*_) may depend on the position of **S**, unique orbital contributions to the MIRC strength can be obtained by calculating the average of the MIRC strength for various orientations of **S** around the ring. This can be done by rotating **S** around the center of the ring with one of its edges along the MIRC vortex as shown in Fig. 1A. The average MIRC strength 〈*I*_*i*_〉 is then obtained as8
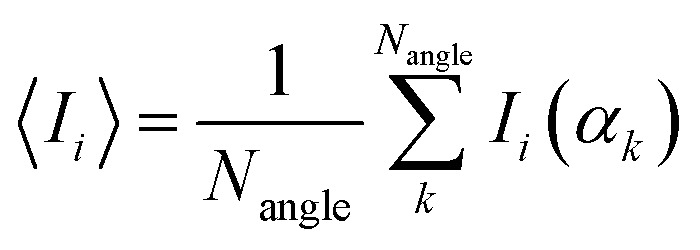
which corresponds to numerical integration around a circle using the trapezoidal rule. We use an integration step of Δ*α* = 1° to ensure high accuracy.

## 3 Computational details

The molecular structures were optimized with Turbomole^71–73^ at the density functional theory (DFT) level using the B3LYP functional,^74,75^ the empirical D4 dispersion correction^76^ and the def2-TZVP^77^ basis sets. The one-electron density matrix and the three perturbed density matrices were obtained by calculating the nuclear magnetic resonance (NMR) shielding constants at the DFT level^78,79^ using the B3LYP^74,75^ functional, which is a popular functional that has been used in many applications. We have also calculated NMR shielding constants with the strong-correlation local hybrid functional (scLH22t).^80,81^ The scLH22t functional was chosen because an accurate magnetizability of the ozone molecule is obtained with it. Further geometry optimizations and calculations of density matrices were performed for C_3_H_3_^+^ to investigate the convergence of the MIRC strength and orbital contributions to the MIRC strengths with respect to the basis-set size. The B3LYP functional and the def2-QZVP, cc-pVTZ, cc-pVQZ, cc-pV5Z*, and cc-pV6Z* basis sets were used.^77,82–84^ Basis functions with a larger angular momentum number than *l* = 4 were removed from the cc-pV5Z and cc-pV6Z basis set because the present implementation cannot handle *h* and higher angular momentum functions.

The MICD susceptibility tensor was calculated with the GIMIC program.^85^ The integration plane begins 8 bohr above the molecular plane and ends 8 bohr below it. In the other direction it begins at the symmetry axis and ends 15 bohr from the axis. The plane is divided into elements whose dimension is 0.05 × 0.05 bohr. We use 9 × 9 Gauss integration points in each element to ensure a high accuracy. The **J**(**r**) vector field was obtained by contracting the tensor with the direction of the external magnetic field. The external magnetic field was aligned along the main symmetry axis. The MIRC strengths were obtained by integrating the **J**(**r**) passing through a plane that cuts through the molecule.^11–13^ The molecular structures and orbitals are visualized with Chemcraft,^86^ and the **J**(**r**) pictures were made with ParaView.^87^ The plots were generated using Matplotlib, a Python library for data visualization.^88^

## 4 Results and discussion

### 4.1 Benzene

The orbital contributions to the MIRC strength calculated for the core, valence σ and π orbitals of benzene are given in Table 1. The total orbital contributions of all orbitals in each irreducible representation of the *C*_6h_ point are divergence free in the limit of complete basis set when using the GIMIC method. The MICD susceptibility calculated with the GIMIC program is practically divergence free when using standard basis sets (def2-TZVP) as seen in Fig. 1B. The MIRC contributions of all orbitals in the irreducible representations are also given in Table 1.

**Table 1:** The average orbital contributions to the MIRC strength (〈*I*_*i*_〉 in nA/T) and the total MIRC strengths of the core, σ, and π orbitals of benzene calculated at the B3LYP and scLH22t levels. The orbital contributions and the contributions of all orbitals in each irreducible representation of the *C*_6h_ point group are compared to the orbital contributions of the isotropic shielding constant (*σ*(0) in ppm) previously calculated at the Hartree–Fock level in the center of the ring using the ipsocentric method.^25^ We also compare to orbital contributions to the *σ*_zz_(0) value calculated at the DFT level using GIAOs^89^

Contribution						
D_6h_	C_6h_	Type	B3LYP	scLH22t	*σ*(0)^25^	*σ*_zz_(0)^89^
1a_1g_	1a_g_	Core	0.06	0.03	0.10	—
1e_1u_	1e_1u_	Core	0.09	0.15	−0.64	—
1e_2g_	1e_2g_	Core	1.19	1.22	0.73	—
1b_2u_	1b_u_	Core	0.77	0.84	−0.06	—
2a_1g_	2a_g_	σ	3.03	3.15	−0.40	14.4
2e_1u_	2e_1u_	σ	4.88	5.26	0.09	11.4
2e_2g_	2e_2g_	σ	4.35	4.56	8.94	8.8
3a_1g_	3a_g_	σ	2.81	2.73	0.34	12.3
2b_2u_	2b_u_	σ	−0.51	−0.36	−2.25	−2.9
3e_1u_	3e_1u_	σ	−3.01	−3.39	−7.47	−14.0
1b_1u_	3b_u_	σ	1.79	1.62	0.01	4.2
1a_2u_	1a_u_	π	4.12	4.14	2.92	12.8
3e_2g_	3e_2g_	σ	−15.16	−15.46	−11.34	−67.6
1e_1g_	1e_1g_	π	7.53	7.59	18.90	23.4
Core			2.12	2.24	0.13	10.8
σ			−1.82	−1.90	−12.06	−33.3
Core + valence σ			0.30	0.34	−11.94	−22.5
π			11.65	11.73	21.83	36.2
	A_g_		5.90	5.91	0.04	26.7^a^
	B_u_		2.05	2.10	−2.30	1.3^a^
	E_1u_		1.96	2.02	−8.02	−2.6^a^
	A_u_		4.12	4.14	2.92	12.8
	E_2g_		−9.62	−9.68	−1.67	−58.8^a^
	E_1g_		7.53	7.59	18.90	23.4
Total			11.95	12.07	9.89	13.7

a The core contributions are not included.

The MIRC strengths (in nA/T) calculated with GIMIC and the isotropic shielding constant in the center of the ring (*σ*(0) in ppm) calculated with the ipsocentric approach^25^ correlate suggesting that similar orbital contributions to the MICD susceptibility are obtained using GIMIC and the ipsocentric method. Orbital contributions to the MICD susceptibility and other magnetic properties of benzene have also been studied using the ipsocentric method,^24,25^ where they found that the HOMO contribution to **J**(**r**) of benzene is diatropic and mainly determines the MIRC strength.^25^ The GIMIC calculations yield a total π contribution of 11.65 nA/T, whereas the core + valence σ contribution is only 0.30 nA/T. These values are close to the ones of 11.7 nA/T and 1.1 nA/T, respectively, which were previously calculated at the Hartree–Fock level with the SYSMOIC program using the ipsocentric approach.^48^

The diatropic π contribution to the MIRC of benzene originates from the π orbitals in the A_u_ and E_1g_ irreducible representations of the *C*_6h_ point group. The orbitals in the E_2g_ irreducible representation sustain a strong paratropic MIRC of −9.62 nA/T, which is almost canceled by contributions of the valence σ and core orbitals in the other irreducible representations of the *C*_6h_ point group. Since the separation into the core and valence σ contributions to the MIRC strength depends on the orientation and position of the integration plane, we compare average contributions that are obtained as described in Section 2.3. The average contribution of the core and valence σ orbitals are 2.12 nA/T and −1.82 nA/T, respectively. The contributions of the valence σ and core orbitals are tiny when the integration plane cuts the ring in the middle of a C–C bond as seen in Fig. 1C.

In Table 1, one can see that all occupied valence orbitals contribute significantly to the MIRC strength. The contribution of the core orbitals is diatropic, whereas the contributions of the valence σ orbitals are both positive and negative. The 〈*I*_*i*_〉 values of the valence orbitals have in general the same sign as the corresponding orbital contributions to *σ*(0), since diatropic orbital contributions to the MIRC result in diamagnetic contribution to *σ*(0) and paratropic orbital contributions to the MIRC result in paramagnetic shielding contributions.^49,50^ Even though the orbital contributions to the MIRC strength and to *σ*(0) have the same trend, the relative size of the contributions significantly differs. The discrepancy is more severe when comparing the formally divergence-free contributions of all orbitals in each irreducible representation. The orbitals in the A_g_ irreducible representation sustain an MIRC of 5.90 nA/T at the B3LYP level, whereas the corresponding *σ*(0) value of 0.04 ppm is tiny.

**Fig. 2:**
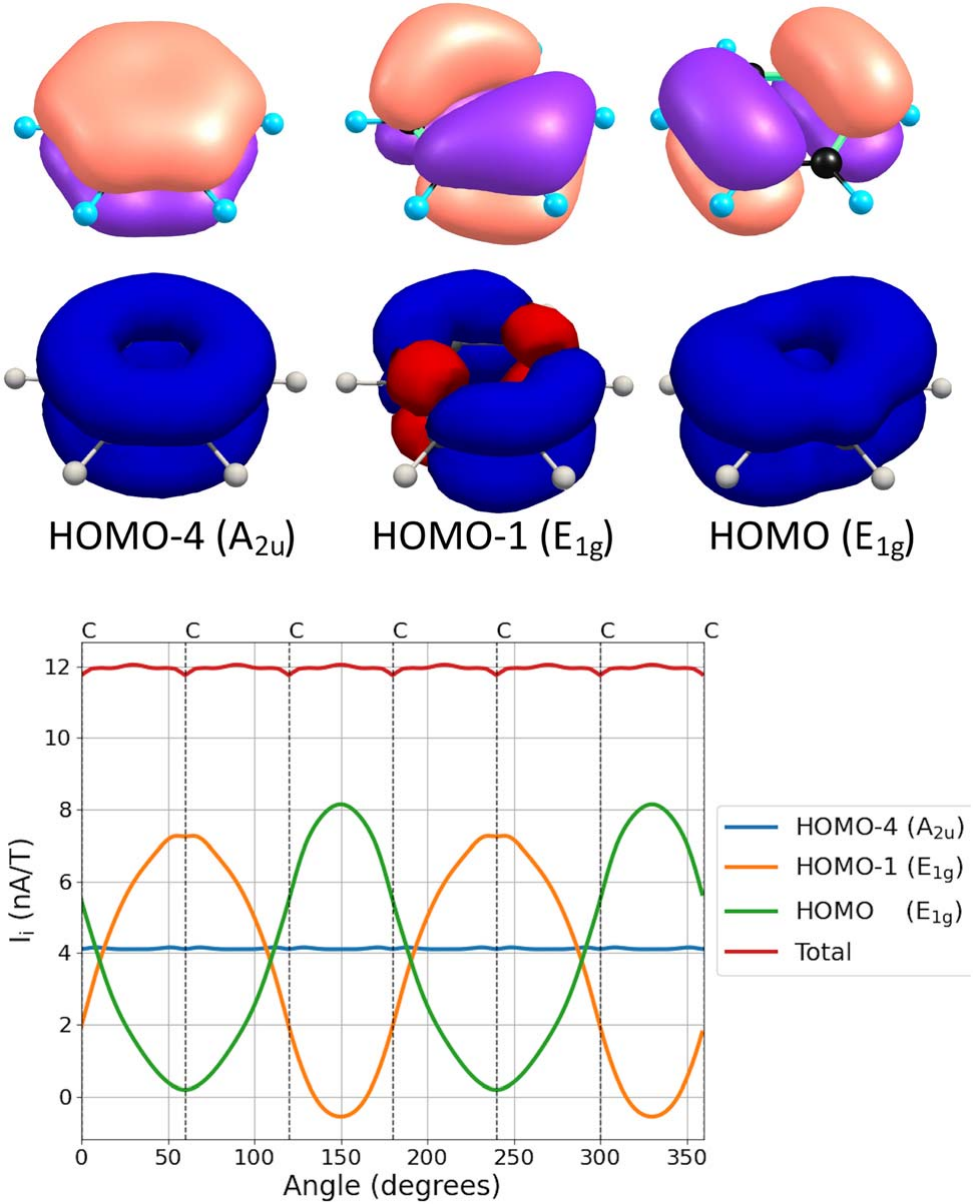
The π orbitals of benzene (top), their MICD (middle) and the dependence of their contributions to the MIRC strength (in nA/T) as a function of the angle of the integration plane (bottom). The orbital pictures are made with ParaView.^87^

For the B_u_ and E_1u_ irreducible representations, the MIRC strength and the *σ*(0) contributions have opposite signs. The MIRC contribution of the orbitals in the E_2g_ irreducible representation is strong and paratropic, whereas the *σ*(0) contribution is weakly paramagnetic. The π contribution to the MIRC strength of the only orbital in the A_u_ irreducible representation is about 35% of the MIRC, which is of the same relative size as the A_u_ contribution to *σ*(0). The MIRC contribution of the two π orbitals in the E_1g_ irreducible representation is about 65% of the MIRC strength, whereas the corresponding contribution to *σ*(0) is a factor of two larger than the total *σ*(0) value. The three π orbitals (HOMO−4, HOMO−1 and HOMO) and their MIRC contributions as a function of the orientation angle of the integration plane are shown in Fig. 2. The MIRC strength of HOMO−4 with respect to the orientation of the integration plane is almost independent of the angle because HOMO−4 is the only orbital in the irreducible representation A_u_ of the group of points *C*_6h_. The oscillating behavior of the contributions of the two π orbitals in E_1g_ and that the sum of them is constant are also seen in Fig. 2. The small waves of the horizontal lines near the nuclei are due to a tiny **J**(**r**) leakage because finite basis sets have been used.^11^

There is in general no clear correlation between the orbital contribution to the MIRC strength and the orbital contributions to the *σ*_zz_(0) and *σ*(0) values of benzene. However, the MIRC strength of the π orbitals and the π-orbital contributions to the *σ*_zz_(0) values correlate. In a study on 43 molecular rings, the best linear correlation was obtained for NICS_π,zz_(*z*) (−*σ*_π,zz_(*z*)) values and MIRC strengths of the π orbitals when *z* is 1.25 Å.^90^

Even though the *σ*(0) contributions are calculated at the Hartree–Fock level using the ipsocentric approach,^48^ the comparison of the MIRC strengths and *σ*(0) values casts a shadow over the ability of NICS calculations to estimate orbital contributions to the MIRC strength. We discuss in the text only MIRC strengths calculated at the B3LYP level, since calculations on aromatic molecules using the scLH22t functional yield MIRC values that agree well with the ones obtained at the B3LYP level. See Table 1.

### 4.2 Borazine

The contributions to the MIRC strength of borazine of all orbitals of each irreducible representation of the *C*_3h_ point group are given in Table 2, whereas the contributions of each occupied orbital are given in the ESI.† The average MIRC contributions of the core, valence σ and π orbitals are also reported in Table 2. The orbital contribution to the MIRC strength of the HOMO is −0.47 nA/T. The HOMO is a doubly degenerate π orbital and the only orbital belonging to the E′′ irreducible representation. For benzene, the HOMO–LUMO transition is translationally allowed, resulting in a strong diatropic contribution to the MIRC of the HOMO, whereas the HOMO–LUMO transition of borazine is translationally and rotationally allowed,^25^ resulting in a weak MIRC contribution. The **J**(**r**) of the HOMO of borazine consists of local vortices circulating around the nitrogen atoms.^91^ The MIRC strength of the HOMO is independent of the orientation of the integration plane because it is the only occupied orbital belonging to the *E*′′ irreducible representation.

**Table 2:** The average contributions to the MIRC strength of borazine (〈*I*_*i*_〉 in nA/T) of all orbitals in each irreducible representation of the *C*_3h_ point group. The calculations were performed at the B3LYP and scLH22t levels. The contributions of the core, valence σ and π orbitals are also given. Orbital contributions are reported in the ESI

Contribution	B3LYP	scLH22t
A′	7.14	7.06
E′	−6.94	−6.87
A′′	3.52	3.50
E′′	−0.46	−0.63
Core	3.04	2.80
Valence σ	−2.85	−2.61
Core + valence σ	0.20	0.19
π	3.06	2.87
Total	3.25	3.06

The MIRC contribution of HOMO−2 is 3.52 nA/T, which is the third π orbital. Borazine is weakly aromatic^91^ sustaining an MIRC of 3.25 nA/T, which is at the borderline between the MIRC strength of aromatic and nonaromatic molecules. Molecules with an absolute MIRC strength of <3 nA/T *i.e.*, 25% of the benzene value can be considered nonaromatic. The average contribution of the core orbitals of 3.04 nA/T almost cancels the contribution of the valence σ orbitals of −2.85 nA/T. The contributions of the core + valence σ orbitals of 0.2 nA/T and of the π orbitals of 3.06 nA/T are close to the ones of 0.3 nA/T and 1.8 nA/T, respectively, which were obtained in SYSMOIC calculations at the Hartree–Fock level using the ipsocentric approach.^48^

### 4.3 1,5-Dibora−2,4-diazabenzene

1,5-Dibora-2,4-diazabenzene (C_2_B_2_N_2_H_6_) is isoelectronic with benzene and aromatic sustaining an MIRC strength of 7.67 nA/T, which is more than 60% of the one for benzene. Since it belongs to the *C*_2v_ point group, *C*_s_ is its highest possible point group in the presence of an external magnetic field. The contributions to the MIRC strength of C_2_N_2_B_2_H_6_ of all orbitals in each irreducible representation of the *C*_s_ point group are given in Table 3, whereas the contributions of each occupied orbital are given in the ESI.† The MICD susceptibility in the A′ (σ) and A′′ (π) irreducible representations is divergence free. The MIRC strength of the π orbitals is 7.59 nA/T. Thus, almost no MIRC is sustained in the σ orbitals. The average MIRC strength of the core orbitals of 2.67 nA/T is canceled by the contributions of the valence σ orbitals. The MIRC strengths of the core and valence σ orbitals depend on the orientation of the integration plane because the orbitals belong to the same irreducible representation of the *C*_s_ point group.

**Table 3:** The average contributions to the MIRC strength of C_2_B_2_N_2_H_6_ (〈*I*_*i*_〉 in nA/T) of the orbitals in the two irreducible representations of the *C*_s_ point group. The calculations were performed at the B3LYP and scLH22t levels. The contributions of the core, valence σ and π orbitals are also given. Orbital contributions are reported in the ESI

Contribution	B3LYP	scLH22t
Core	2.67	2.52
Valence σ	−2.59	−2.42
A′ (core + valence σ)	0.08	0.10
A′′ (π)	7.59	7.55
Total	7.67	7.65

### 4.4 Porphin

For porphin, the average contribution of the core orbitals of 11.22 nA/T is almost canceled by the contribution of the valence σ orbitals of −10.75 nA/T. The resulting contribution of the core + valence σ orbitals of 0.47 nA/T is much smaller than the average contribution of 27.2 nA/T of the π orbitals. The total MICD strength is 27.68 nA/T. The contributions to the MIRC strength of porphin of all orbitals in each irreducible representation of the *C*_2h_ point group are given in Table 4. The contributions of the core, valence σ, and π orbitals are also reported.

**Table 4:** The average contributions to the MIRC strength (in nA/T) of porphin, tetraoxa-isophlorin, 1,4-cyclohexadiene and cyclobutadiene of the orbitals in each irreducible representation of the *C*_2h_ point group. The calculations were performed at the B3LYP and scLH22t levels. The contributions of the core, valence σ and π orbitals are also given. Orbital contributions are reported in the ESI

Contribution	Porphin		Isophlorin		1,4-Cyclohexadiene		Cyclobutadiene	
	B3LYP	scLH22t	B3LYP	scLH22t	B3LYP	scLH22t	B3LYP	scLH22t
A_g_	0.06	0.14	−0.26	−0.34	−4.08	−4.16	8.89	8.85
B_g_	12.50	12.65	−76.80	−60.45	3.62	3.46	−19.88	−20.56
A_u_	14.70	14.90	13.62	12.95	−4.41	−4.13	3.84	3.85
B_u_	0.41	0.48	−0.56	−0.59	4.23	4.26	−12.79	−12.91
Core	11.22	10.56	10.86	10.31	2.03	2.14	1.32	1.41
σ	−10.75	−9.94	−11.69	−11.24	−1.88	−2.04	−5.22	−5.47
Core + valence σ	0.62	−0.83	−0.93	0.15	0.11	−3.90	−4.06	
π	27.20	27.55	−63.17	−47.50	−0.79	−0.67	−16.05	−16.71
Total	27.68	28.17	−64.00	−48.44	−0.64	−0.56	−19.95	−20.76

About half of the π contribution to the MIRC strength (14.70 nA/T) originates from the orbitals in the A_u_ irreducible representations of the *C*_2h_ point group and the contribution of the B_g_ orbitals is 12.50 nA/T. The averaged contributions of the individual orbitals in the ESI† show that nine π orbitals contribute more than 1 nA/T to the total MIRC. The individual MIRC contribution of these π orbitals is almost the same for seven of them (∼3.5 nA/T). The only exceptions are the contributions of HOMO of 2.20 nA/T and of HOMO−1 of 1.28 nA/T. The sum of their contributions is also ∼3.5 nA/T. The orbital contributions of four of the π orbitals are tiny, that is, in the range of ± 1nA/T. The contributions of HOMO−2 and HOMO−3 are −1.01 nA/T and −0.01 nA/T, respectively. A previous study of orbital contributions to the **J**(**r**) of porphin using the ipsocentric method suggested that four frontier π orbitals contribute to the MIRC strength,^31^ which does not agree with the results obtained in our study. The total MIRC strength is almost entirely determined by contributions of the π orbitals. However, many energetically low-lying π orbitals contribute to the total MIRC.

The diatropic contribution to the MIRC of the HOMO can be explained by the translationally allowed transition from HOMO (b_1u_) to LUMO (b_3g_), whereas the diatropic contributions of the energetically low-lying π (b_1u_) orbitals using selection rules may involve transitions to many virtual orbitals belonging to the same irreducible representation as the LUMO.

### 4.5 Nonaromatic molecule

The absence of continuous electron delocalization is generally the reason why molecular rings are nonaromatic. Here, we study 1,4-cyclohexadiene as an example of a nonaromatic molecular ring. The electron delocalization of 1,4-cyclohexadiene ring is disrupted by the out-of-plane hydrogen atoms at the two sp^3^-hybridized carbon atoms. The MIRC strengths of the σ and π orbitals of 1,4-cyclohexadiene can be separated because its molecular structure belongs to the *D*_2h_ point group. In the presence of a magnetic field along the main symmetry axis, the core and valence σ orbitals belong to the A_g_ and B_u_ irreducible representations of the *C*_2h_ point group. The sum of their contributions to the MIRC strength is 0.15 nA/T. An MIRC strength of the π orbitals of −0.79 nA/T is obtained as the sum of the contributions of the orbitals in the A_u_ and B_g_ irreducible representations. Since 1,4-cyclohexadiene does not sustain any strong MIRC in the σ nor in the π orbitals it is nonaromatic, even though the transitions between occupied and unoccupied frontier orbitals are translationally allowed. The MIRC strengths of the orbitals in each irreducible representation are given in Table 4 where we also report the average contributions of the core, valence σ and π orbitals. Orbital contributions are reported in the ESI.†

### 4.6 Antiaromatic molecules

Tetraoxa-isophlorin is a strongly antiaromatic porphyrinoid. Previous studies showed that it has a large magnetic transition moment from the electronic ground state to the lowest excited state resulting in a significant paramagnetic contribution to the magnetizability.^92,93^ Hexadehydro[12]annulene is also an antiaromatic molecule sustaining a strong paratropic MIRC.^94–96^ The average contributions to the MIRC strength of tetraoxa-isophlorin for all orbitals in the irreducible representations of the *C*_2h_ point group are reported in Table 4. The MIRC strength of hexadehydro[12]annulene for all orbitals in the irreducible representations of the *C*_3h_ point group are given in Table 5. Orbital contributions are reported in the ESI.†

**Table 5:** The average contributions to the MIRC strength (in nA/T) of hexadehydro[12]annulene (HDH[12]A), C_3_H_3_^+^ and C_3_H_6_ of the orbitals in each irreducible representation of the *C*_3h_ point group. The calculations were performed at the B3LYP and scLH22t levels. The contributions of the core, valence σ, and π orbitals are also given. Orbital contributions are reported in the ESI

	HDH[12]A		C_3_H_3_^+^		C_3_H_6_	
	B3LYP	scLH22t	B3LYP	scLH22t	B3LYP	scLH22t
A′	−0.01	0.14	3.33	3.35	3.02	3.07
E′	−0.02	−0.26	3.14	3.04	6.42	6.42
A′′	−36.86	−31.05	3.90	3.92	2.84	2.81
E′′	12.77	12.20	—	—	−2.36	−2.30
Core	11.18	9.26	1.07	1.30	0.85	0.79
σ	−11.22	−9.40	5.40	5.09	8.59	8.70
Core + σ	−0.04	−0.14	6.47	6.40	9.44	9.49
π	−24.10	−18.86	3.90	3.92	0.49	0.50
Total	−24.13	−18.99	10.37	10.32	9.92	9.99

The average contributions to the MIRC of the core + valence σ orbitals are nearly zero. The total MIRC strength originates mainly from the π orbitals, which belong to the B_g_ and A_u_ irreducible representations for tetraoxa-isophlorin, and to the A′′ and E′′ irreducible representations for hexadehydro[12]annulene,.

The contributions of the individual orbitals show that the MIRC strength of nine π orbitals of tetraoxa-isophlorin is diatropic. Each of them contributes more than 1.5 nA/T to the total MIRC strength. Their total diatropic MIRC strength is 23.81 nA/T. The diatropic contribution of four other π orbitals is 2.22 nA/T. The total contribution of all π orbitals except one is 26.03 nA/T, which is almost as large as for porphin. The large paratropic contribution of −89.97 nA/T of the HOMO calculated at the B3LYP level makes tetraoxa-isophlorin antiaromatic. Calculations using the scLH22t functional yield a HOMO contribution of −73.54 nA/T showing the importance of significant long-ranged Hartree–Fock exchange in the functional to properly describe magnetic response of strongly antiaromatic molecules, whereas for aromatic molecules the MIRC strengths obtained with the two functionals are almost equal.

Five of the six π orbitals of hexadehydro[12]annulene sustain a total diatropic MIRC of 16.67 nA/T, whereas the MIRC contribution of the HOMO is −40.76 nA/T making the molecule antiaromatic. The strong paratropic MIRC contribution of the HOMO can be explained by using symmetry selection rules. The 
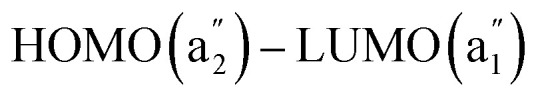
 transition of hexadehydro[12]annulene is purely rotationally allowed as also for the HOMO (b_2g_)–LUMO (b_3g_) transition of tetraoxa-isophlorin.

The planar structure of cyclooctatetraene (COT), which belongs to the *D*_4h_ point group, is reduced to the *C*_4h_ point group in the presence of an external magnetic field along the main symmetry axis. The average contributions to the MIRC strength of COT of all orbitals in each irreducible representation are given in Table 6. The average contributions of the core, valence σ, and π orbitals are also given. Orbital contributions are reported in the ESI.†

The average contributions to the MIRC strength (〈*I*_*i*_〉 in nA/T) of planar cyclooctatraene for all orbitals in the irreducible representations of the *C*_4h_ point group and of the bent cyclooctatraene for all orbitals in the irreducible representations of the *S*_4_ point group are given. The calculations were performed at the B3LYP and scLH22t levels. Average contributions of the core, valence σ and π orbitals are also given. Orbital contributions are reported in the ESI

**Table:** 

Contribution (*C*_4h_)	B3LYP	scLH22t
A_g_	6.45	6.46
B_g_	−5.65	−5.83
E_g_	7.48	7.30
A_u_	4.10	4.11
B_u_	−51.68	−46.47
E_u_	−1.11	−1.17
Core	4.06	3.74
σ	−4.37	−4.28
Core + valence σ	−0.30	−0.54
π	−40.10	−35.06
Total	−40.41	−35.60

**Table:** 

Contribution (*S*_4_)	B3LYP	scLH22t
A	−7.47	−6.37
B	−2.02	−2.04
E	6.67	6.46
Total	−2.82	−1.96

Planar COT is antiaromatic and characterized by a strong paratropic HOMO contribution of −51.68 nA/T to the MIRC strength, which can be explained by the rotationally allowed HOMO (b_2u_)–LUMO (b_1u_) transition.^97^ The three other π orbitals sustain diatropic contributions to the MIRC strength. The sum of their contributions is 11.58 nA/T, which is close to the MIRC strength of benzene. The core + valence σ contribution of only −0.30 nA/T can be ignored. Planar COT is strongly antiaromatic sustaining a paratropic MIRC of −40.41 nA/T at the B3LYP level. The MIRC strength of −35.60 nA/T calculated with the scLH22t functional is slightly smaller.

The MIRC contributions of the core + valence σ and π orbitals of planar cyclooctatetraene have been previously calculated at the Hartree–Fock level with the SYSMOIC program. The obtained core + valence σ contribution of −1.1 and the π contribution of −18.0 nA/T also show that the π contribution dominates making the molecule antiaromatic.^48^

Bent cyclooctatetraene belonging to the *D*_2d_ point group is energetically more stable than the planar structure.^98^ The average MIRC contribution of the HOMO of −12.77 nA/T for the bent cyclooctatetraene structure shows that bending the molecule significantly reduces the HOMO contribution to the MIRC strength. The total MIRC strength of the bent COT is only −2.82 nA/T suggesting that it is nonaromatic. The paratropic MIRC contribution of the HOMO is the largest one. Many other orbitals contribute significantly. The average contribution of the core orbitals is 2.77 nA/T. Orbital MIRC strengths are reported in the ESI.† The divergence-free contributions of all orbitals in each irreducible representation of the *S*_4_ point group are given in Table 6. The spatial distributions of the **J**(**r**) of the HOMO of the planar and bent cyclooctatetraene are shown in the ESI.†

### 4.7 Strain

Molecular properties of planar ring-shaped molecules, whose ∠CCC angles significantly smaller than the standard ∠CCC angle of sp^2^ hybridized hydrocarbons, differ from those of ordinary molecular rings due to the ring strain.^99^ The strain leads to delocalization of the electrons in the σ orbitals and often to σ aromaticity. However, all strained molecular rings do not exhibit σ aromaticity.

We study orbital contributions to the **J**(**r**) of the planar cyclopropenium cation (C_3_H_3_^+^) and cyclobutadiene (C_4_H_4_) because their magnetic properties have significant contributions of the σ orbitals. Previous studies of the delocalization energy of the C_3_H_3_^+^ and C_4_H_4_ suggested that C_3_H_3_^+^ is σ + π aromatic and that C_4_H_4_ is σ + π antiaromatic.^100,101^ Calculations of the orbital contributions to the MIRC strength of C_4_H_4_ show that 20% of the MIRC strength is sustained by the core + valence σ orbitals. Since the core contribution is diatropic, the paratropic MIRC contribution of valence σ orbitals is even 25% of the total MIRC strength. The MIRC strength of all orbitals of C_4_H_4_ belonging to the irreducible representations of the *C*_2h_ point group are given in Table 4. The total MIRC of the antiaromatic C_4_H_4_ is −19.95 nA/T at the B3LYP level and −20.76 nA/T at the scLH22t level. The HOMO (b_2g_) → LUMO (b_3g_) and the HOMO−1 (b_2u_) → LUMO+1 (b_3u_) transitions of C_4_H_4_ are rotationally allowed leading to σ + π antiaromaticity. Thus, a significant paratropic MIRC contribution of −12.25 nA/T arises from the HOMO−1 σ orbital due to the strain.

The core + valence σ contribution to the MIRC strength of C_3_H_3_^+^ is 6.47 nA/T, which is even larger than the π contribution of 3.90 nA/T. Since the average core contribution is 1.07 nA/T, the MIRC is dominated by the valence σ contribution. The MIRC contributions of C_3_H_3_^+^ for all orbitals of the irreducible representations of the *C*_3h_ point group are given in Table 5. The total MIRC strength of 10.37 nA/T is almost as larger as the MIRC strength of benzene. All orbitals except the HOMO sustain a diatropic MIRC. The MIRC of the HOMO is unexpectedly paratropic with a MIRC strength of −3.49 nA/T because the HOMO (e′) to LUMO+1 (e′) transition is rotationally allowed.

Previous computational studies on cyclopropane^54,56^ (C_3_H_6_) suggested that it is not σ aromatic, whereas other MICD susceptibility studies on C_3_H_6_ yielded a strong MIRC in the σ orbitals and a weak MIRC contribution in the π orbitals.^48,55^ The present calculations of the orbital contributions to the MIRC of C_3_H_6_ confirm that the MIRC is mainly sustained in the σ orbitals as shown in Table 5. Even though the total MIRC of C_3_H_3_^+^ (10.37 nA/T) and of C_3_H_6_ (9.92 nA/T) are almost equal, the contribution of the core + valence σ orbitals to the MIRC strength of C_3_H_6_ of 9.44 nA/T dominates. It is an order of magnitude larger than the π-orbital contribution of 0.49 nA/T. The contribution of the HOMO to the MIRC strength of C_3_H_6_ is 1.57 nA/T. The HOMO (e′) → LUMO+2 (e′) transition is translationally allowed, whereas the HOMO (e′) → LUMO 
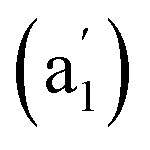
 transition is both translationally and rotationally forbidden. The contributions to the MIRC strength of core + valence σ and π orbitals obtained in this study are also in good agreement with the previously reported ones of 9.7 nA/T and 0.5 nA/T, respectively, which were obtained using the SYSMOIC program.^48^

The extended Hückel rule for σ orbitals^51^ suggests that C_4_H_4_ is σ antiaromatic and C_3_H_3_^+^ is σ aromatic, since C_4_H_4_ has 4*n* core + valence σ electrons and C_3_H_3_^+^ has 4*n* + 2 core + valence σ electrons.

**Fig. 3:**
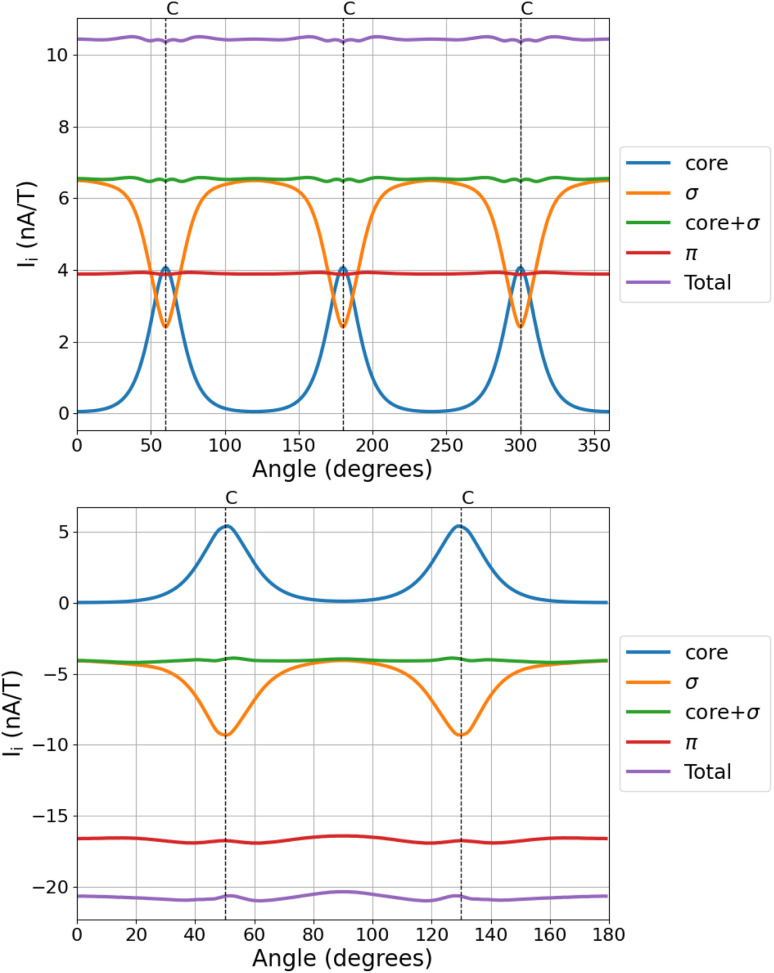
The angular dependence of the contributions of the core, valence σ, and π orbitals of C_3_H_3_^+^ (top) and of cyclobutadiene (bottom). The MIRC strengths of C_3_H_3_^+^ were calculated using the def2-QZVP basis sets to show that the divergence is smaller when using larger basis sets. The positions of the nuclei are indicated by the vertical dashed lines.

The angular dependence of the MIRC contributions of the core, valence σ, and π orbitals of C_3_H_3_^+^ and C_4_H_4_ is shown in Fig. 3. The core contribution is diatropic for all directions of the integration plane and vanishes when the plane passes through the middle of the C–C bond. The core contribution increases in the vicinity of the nuclei, where the valence σ contribution to the MIRC strength decreases. The sum of the two contributions is independent of the orientation angle of the integration plane. The core + valence σ contribution is tiny for most of the studied molecules, whereas for C_3_H_3_^+^ and C_4_H_4_ the sum of the two contributions is independent of the angle of the integration plane and it is of the same size as the π contribution to the MIRC strength.

### 4.8 Basis-set effects

The MICD susceptibility is formally divergence free. The charge conservation condition of the orbital contributions to **J**(**r**) is fulfilled when considering all orbitals in a given irreducible representation of the point group of the molecular structure in the presence of an external magnetic field. However, the condition is not fulfilled in every point in space when finite basis sets are used in the GIMIC calculation. Even though one can correct the divergence of the MICD susceptibility,^45^ it is generally not necessary because the divergence of the MICD susceptibilities obtained in GIMIC calculations is small and does not affect most interpretations. The MIRC strengths were calculated as a function of the orientation angle of the integration plane as discussed in Section 2.3. The difference between the maximum and minimum values of the total MIRC strength of C_3_H_3_^+^ and the orbital contributions to the MIRC strength of all orbitals in each irreducible representation of the *C*_3h_ point group are reported in Table 7. The angular dependence of the MIRC strength decreases when using systematically larger basis sets from cc-pVTZ to cc-pV6Z, while the mean values are almost converged with the triple-*ζ* quality basis sets. The orbital contributions as a function of the orientation angle of the integration plane are not constant even with the largest basis set suggesting that larger basis sets than cc-pV6Z must be employed to obtain a MICD susceptibility that is divergence free in all points of space. The cc-pV6Z basis sets are large but they are probably not flexible enough near the nuclei.

**Table 7:** The mean (nA/T) and the range (*Δ*, nA/T) of the MIRC strength, calculated as the difference between the minimum and maximum values, are obtained from the angular dependence of the MIRC contributions of C_3_H_3_^+^ for all orbitals in each irreducible representation of the *C*_3h_ point group. The MIRC strengths are calculated at the B3LYP level using the cc-pVTZ, cc-pVQZ, cc-pV5Z and cc-pV6Z basis sets

	cc-pVTZ		cc-pVQZ		cc-pV5Z		cc-pV6Z	
	Mean	*Δ*	Mean	*Δ*	Mean	*Δ*	Mean	*Δ*
A′	3.33	0.29	3.32	0.29	3.32	0.21	3.32	0.16
E′	3.15	0.92	3.21	0.47	3.22	0.27	3.22	0.17
A′′	3.90	0.10	3.91	0.05	3.91	0.03	3.91	0.01
Total	10.37	0.81	10.44	0.27	10.44	0.11	10.45	0.04

## 5 Summary and conclusions

We have developed and implemented a novel approach in the GIMIC program for calculating contributions of occupied orbitals to the magnetically induced current density (MICD) susceptibility tensor. The method has been applied to various aromatic, nonaromatic and antiaromatic monocyclic molecules. The orbital contributions to the MIRC strength of benzene, borazine, 1,4-cyclohexadiene, 1,5-dibora-2,4-diazabenzene, the cyclopropenium cation, cyclobutadiene, planar cyclooctatetraene, bent cyclooctatetraene, hexadehydro[12]annulene, porphin and tetraoxa-isophlorin have been thoroughly investigated. The strength of the magnetically induced ring current (MIRC) has been obtained by numerically integrating the MICD susceptibility passing through an integration plane that crosses the molecular ring using accurate integration grids. We have used various orientations of the integration plane to assess how well the charge-conservation condition of the MICD susceptibility is fulfilled. The present results agree with previously reported data that have been calculated for some of the studied molecules using alternative methods. The present work is more comprehensive presenting for the first time detailed investigations of the orbital contributions to the MICD susceptibility and the MIRC of the eleven studied molecules.

The orbital contributions to the MICD susceptibility are not divergence free implying that the orbital contributions to the MIRC strength vary around the molecular ring. Unique average orbital contributions to the MIRC strength were obtained by calculating the MIRC strength for various orientations of the integration plane. Average MIRC strengths were obtained by numerically integrating over the orientation angle around the whole ring. The total MIRC and the MIRC contributions of all orbitals belonging to a given irreducible representation of the molecular point group in the presence of an external magnetic field are formally divergence free. The charge leakage of the MICD susceptibility calculated using the GIMIC method is tiny and can in most applications be ignored. The leakage decreases when increasing the size of the basis set and vanishes in the complete basis-set limit.

For planar antiaromatic molecules, the MIRC contribution of the HOMO is strongly paratropic and much larger than the contributions of the other orbitals, which is not the case for aromatic molecules. The total MIRC of aromatic molecules consists of orbital contributions of many orbitals. The orbital contributions to the MIRC strengths of C_4_H_4_, C_3_H_3_^+^ and C_3_H_6_ showed that the ring strain leads to a strong MIRC strength in an energetically high-lying σ orbital. The MIRC of the σ orbital of C_4_H_4_ is paratropic and for C_3_H_3_^+^ and C_3_H_6_ it is diatropic.

The presented algorithm works well for molecules whose ground-state wave function is dominated by a single Slater determinant. The size of the molecules that can be studied is limited only by the calculations of the NMR shielding constants. Since molecular magnetic properties can be obtained with the GIMIC program by numerical integration of the MICD susceptibility multiplied by the vector potential of the magnetic property, the present work also opens new possibilities for investigating orbital contributions to molecular magnetic properties.

## Data availability

The data supporting this article have been included as part of the ESI.† Density functional theory calculations were performed with Turbomole version 7.8. The Turbomole webpage is https://www.turbomole.org/. GIMIC, version 2.0 can be freely downloaded from https://github.com/qmcurrents/gimic and https://zenodo.org/record/8180435. The new features of the GIMIC program are not yet available to everyone. ParaView can be downloaded from https://www.paraview.org/.

## Author contributions

RTN implemented the methods, performed the calculations and prepared the pictures. DS proposed the project and developed the theory together with RTN. RTN and DS wrote the first version of the manuscript. All authors contributed to writing the article.

## Conflicts of interest

There are no conflicts to declare.

## Supplementary Material

SC-016-D5SC00627A-s001

SC-016-D5SC00627A-s002
