# Supplementary material for: Orbital contributions to magnetically induced current densities using gauge-including atomic orbitals
Source: Chem Sci. 2025 Apr 1;16(18):8040–52. doi: 10.1039/d5sc00627a (PMC11976446; doi:10.1039/d5sc00627a)
Supplement: SC-016-D5SC00627A-s002 [file SC-016-D5SC00627A-s002.pdf]

Supplementary Information (SI) for Chemical Science.
This journal is © The Royal Society of Chemistry 2025

Supplementary Materials

Orbital Contributions to Magnetically Induced Current Densities Using Gauge-Including Atomic Orbitals

Rinat T. Nasibullin[a], Maria Dimitrova[a], Rashid R. Valiev[a], and Dage Sundholm[a]

[a]Department of Chemistry, University of Helsinki, P.O. Box 55, FIN-00014 Helsinki, Finland.

Contents

1 Visualization of magnetically induced current densities 2

2 Average orbital contributions to the MIRC 3

3 The angular dependence of the MIRC strength of the core, valence σ and π orbitals. 12

4 The angular dependence of the MIRC strength of all orbitals of each irreducible representation 16

5 Optimized geometries of the studied molecules. 22

1 Visualization of magnetically induced current densities

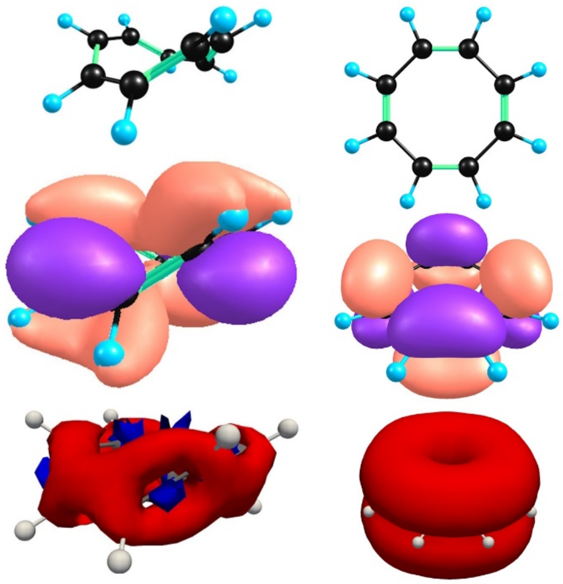

Figure S1: The molecular structure of the bent (right) and planar (left) cyclooctatetraene (top), their HOMO (middle) and the MICD of the HOMO (bottom).

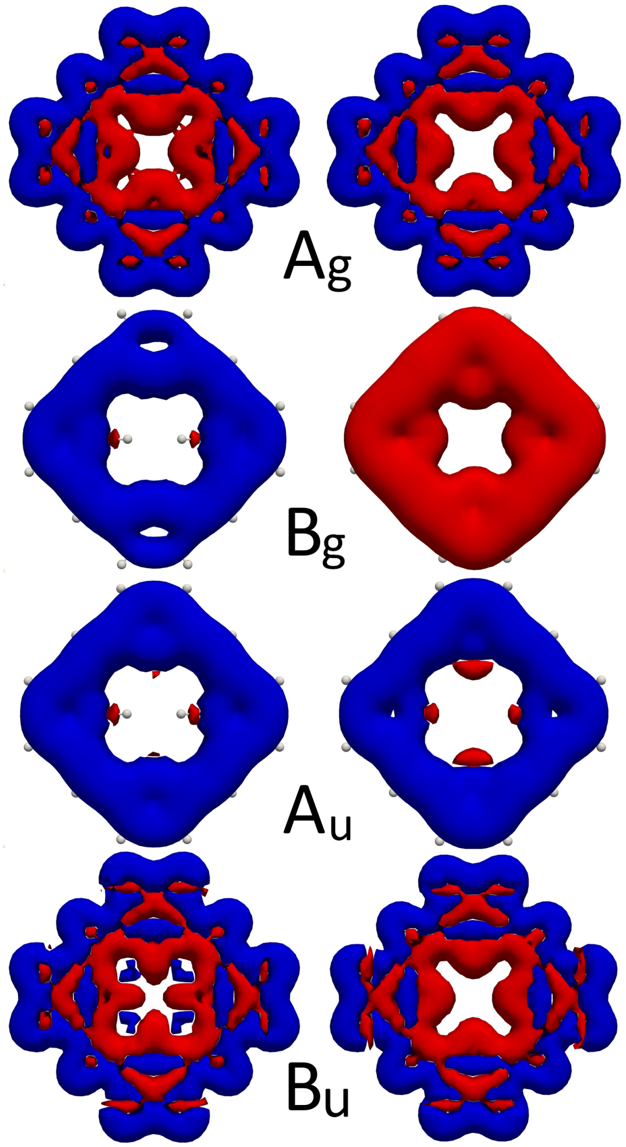

Figure S2: The MICD of all orbitals in the A_g, B_g, A_u, and B_u irreducible representations of the C_{2h} point group of porphin (left) and tetraoxa-isophlorin (right).

2 Average orbital contributions to the MIRC

Table S1: The average orbital contributions to the MIRC strength ($\langle I_i \rangle$ in nA/T) of borazine. The calculations were performed at the B3LYP and scLH22t levels. Orbital types, occupation numbers and irreducible representations are also given.

№	Orbital	Occ.	Type	B3LYP	scLH22t
1	$1e'$	4.0	core	1.70	1.24
2	$1a'_1$	2.0	core	0.53	0.42

Continued on next page

№	Orbital	Occ.	Type	B3LYP	scLH22t
3	$2e'$	4.0	core	0.46	0.70
4	$2a'_1$	2.0	core	0.35	0.44
5	$3a'_1$	2.0	σ	2.67	2.60
6	$3e'$	4.0	σ	5.36	4.78
7	$4a'_1$	2.0	σ	1.83	1.85
8	$4e'$	4.0	σ	1.18	3.27
9	$5e'$	4.0	σ	-3.86	-3.69
10	$1a'_2$	2.0	σ	1.35	1.30
11	$5a'_1$	2.0	σ	0.40	0.45
12	$1a''_2$	2.0	π	3.52	3.50
13	$6e'$	4.0	σ	-11.81	-13.17
14	$1e''$	4.0	π	-0.47	-0.63

Table S2: The average orbital contributions to the MIRC strength (in nA/T) of $C_2B_2N_2H_6$. The calculations were performed at the B3LYP and scLH22t levels. Orbital types and irreducible representations are also given.

№	Orbital	Type	B3LYP	scLH22t
1	$1a_1$	core	0.44	0.34
2	$2a_1$	core	0.74	0.55
3	$1b_1$	core	0.43	0.36
4	$3a_1$	core	0.48	0.44
5	$2b_1$	core	0.25	0.38
6	$4a_1$	core	0.33	0.45
7	$5a_1$	σ	2.57	2.50
8	$6a_1$	σ	2.61	2.45
9	$3b_1$	σ	2.25	2.53
10	$7a_1$	σ	1.68	1.52
11	$8a_1$	σ	1.43	1.60
12	$4b_1$	σ	1.12	2.19
13	$9a_1$	σ	-0.67	-0.67
14	$5b_1$	σ	-0.09	-1.04
15	$1b_2$	π	3.58	3.61
16	$10a_1$	σ	-0.39	0.27
17	$6b_1$	σ	-2.08	-1.37
18	$7b_1$	σ	-4.57	-5.18
19	$11a_1$	σ	-6.45	-7.22
20	$2b_2$	π	2.54	2.55
21	$1a_2$	π	1.47	1.39

Table S3: The average orbital contributions to the MIRC strength ($\langle I_i \rangle$ in nA/T) of porphin. The calculations were performed at the B3LYP and scLH22t levels. Orbital types and irreducible representations are also given.

№	Orbital	Type	B3LYP	scLH22t
1	$1b_{3u}$	core	0.45	0.32
2	$1a_g$	core	0.48	0.36
3	$2a_g$	core	0.45	0.36
4	$1b_{2u}$	core	0.40	0.31
5	$1b_{1g}$	core	0.64	0.58
6	$2b_{2u}$	core	0.64	0.6
7	$2b_{3u}$	core	0.46	0.55
8	$3a_g$	core	0.49	0.62
9	$3b_{3u}$	core	0.74	0.68
10	$2b_{1g}$	core	0.73	0.65
11	$3b_{2u}$	core	0.49	0.61
12	$4a_g$	core	0.49	0.64
13	$3b_{1g}$	core	0.65	0.52
14	$4b_{3u}$	core	0.52	0.38
15	$4b_{2u}$	core	0.48	0.34
16	$5a_g$	core	0.29	0.12
17	$5b_{3u}$	core	0.08	0.07
18	$6a_g$	core	0.08	0.07
19	$4b_{1g}$	core	0.64	0.66
20	$5b_{2u}$	core	0.63	0.66
21	$6b_{2u}$	core	0.08	0.06
22	$7a_g$	core	0.08	0.06
23	$5b_{1g}$	core	0.62	0.66
24	$6b_{3u}$	core	0.62	0.67
25	$8a_g$	σ	2.81	2.63
26	$7b_{3u}$	σ	2.59	2.37
27	$7b_{2u}$	σ	2.97	2.82
28	$9a_g$	σ	3.00	2.94
29	$6b_{1g}$	σ	2.84	2.88
30	$8b_{2u}$	σ	2.63	2.77
31	$10a_g$	σ	2.12	2.1
32	$8b_{3u}$	σ	2.29	2.21
33	$9b_{3u}$	σ	2.73	2.9
34	$9b_{2u}$	σ	2.10	2.08
35	$11a_g$	σ	2.06	1.99
36	$12a_g$	σ	2.54	2.61
37	$7b_{1g}$	σ	1.31	1.59
38	$10b_{2u}$	σ	0.93	1.12
39	$10b_{3u}$	σ	1.32	1.41
40	$13a_g$	σ	1.87	2.02

Continued on next page

№	Orbital	Type	B3LYP	scLH22t
41	8b$_{1g}$	σ	0.91	1.09
42	11b$_{3u}$	σ	1.89	1.94
43	11b$_{2u}$	σ	0.79	0.93
44	9b$_{1g}$	σ	-0.57	0.09
45	14a$_{g}$	σ	2.18	2.19
46	12b$_{3u}$	σ	0.44	0.67
47	10b$_{1g}$	σ	-0.56	-0.19
48	12b$_{2u}$	σ	0.78	1.86
49	15a$_{g}$	σ	2.15	1.89
50	13b$_{2u}$	σ	0.81	0.56
51	13b$_{3u}$	σ	-0.54	0.1
52	16a$_{g}$	σ	1.46	0.79
53	14b$_{3u}$	σ	1.02	0.64
54	11b$_{1g}$	σ	-2.68	-2.21
55	17a$_{g}$	σ	0.49	0.57
56	1b$_{1u}$	π	3.57	3.6
57	1b$_{2g}$	π	3.41	3.43
58	14b$_{2u}$	σ	-1.65	-2.24
59	15b$_{3u}$	σ	-2.08	-1.89
60	18a$_{g}$	σ	-3.42	-2.78
61	12b$_{1g}$	σ	-2.66	-2.51
62	1b$_{3g}$	π	3.53	3.55
63	15b$_{2u}$	σ	-3.81	-3.71
64	2b$_{1u}$	π	3.34	3.38
65	19a$_{g}$	σ	-6.38	-5.94
66	16b$_{3u}$	σ	-4.14	-3.75
67	16b$_{2u}$	σ	-7.37	-7.34
68	13b$_{1g}$	σ	-5.56	-5.45
69	14b$_{1g}$	σ	-7.45	-9.03
70	17b$_{3u}$	σ	-8.07	-9.03
71	1a$_{u}$	π	3.58	3.59
72	2b$_{3g}$	π	3.20	3.23
73	2b$_{2g}$	π	3.42	3.36
74	3b$_{1u}$	π	0.75	0.77
75	17b$_{2u}$	σ	-0.80	-1.19
76	3b$_{2g}$	π	-0.05	0.04
77	20a$_{g}$	σ	-2.05	-2.43
78	4b$_{1u}$	π	-0.01	-0.12
79	3b$_{3g}$	π	-1.01	-0.96
80	2a$_{u}$	π	1.28	1.32
81	5b$_{1u}$	π	2.20	2.36

Table S4: The average orbital contributions to the MIRC strength ($\langle I_i \rangle$ in nA/T) of tetraoxa-isophlorin. The calculations were performed at the B3LYP and scLH22t levels. Orbital types and irreducible representations are also given.

№	Orbital	Type	B3LYP	scLH22t
1	$1b_{2u}$	core	0.32	0.26
2	$1a_g$	core	0.35	0.28
3	$1b_{3u}$	core	0.36	0.30
4	$2a_g$	core	0.40	0.32
5	$1b_{1g}$	core	0.70	0.62
6	$2b_{2u}$	core	0.71	0.65
7	$2b_{3u}$	core	0.40	0.52
8	$3a_g$	core	0.44	0.60
9	$2b_{1g}$	core	0.71	0.62
10	$3b_{3u}$	core	0.71	0.64
11	$3b_{2u}$	core	0.49	0.58
12	$4a_g$	core	0.52	0.65
13	$4b_{3u}$	core	0.09	0.07
14	$5a_g$	core	0.08	0.07
15	$3b_{1g}$	core	0.62	0.67
16	$4b_{2u}$	core	0.61	0.67
17	$4b_{1g}$	core	0.63	0.50
18	$5b_{2u}$	core	0.48	0.34
19	$5b_{3u}$	core	0.53	0.38
20	$6a_g$	core	0.26	0.08
21	$6b_{2u}$	core	0.08	0.08
22	$7a_g$	core	0.08	0.08
23	$5b_{1g}$	core	0.65	0.66
24	$6b_{3u}$	core	0.64	0.66
25	$8a_g$	σ	2.65	2.40
26	$7b_{2u}$	σ	2.52	2.27
27	$7b_{3u}$	σ	2.60	2.30
28	$9a_g$	σ	2.65	2.38
29	$6b_{1g}$	σ	2.72	2.80
30	$8b_{3u}$	σ	2.35	2.48
31	$10a_g$	σ	2.29	2.37
32	$8b_{2u}$	σ	2.44	2.56
33	$11a_g$	σ	2.23	2.31
34	$9b_{3u}$	σ	2.62	2.74
35	$9b_{2u}$	σ	2.39	2.46
36	$12a_g$	σ	2.38	2.44
37	$7b_{1g}$	σ	1.17	1.35
38	$10b_{3u}$	σ	0.74	0.81
39	$10b_{2u}$	σ	0.85	1.07
40	$8b_{1g}$	σ	1.33	1.42

Continued on next page

№	Orbital	Type	B3LYP	scLH22t
41	9b$_{1g}$	σ	-1.31	0.75
42	11b$_{2u}$	σ	0.68	0.85
43	11b$_{3u}$	σ	0.68	-0.57
44	13a$_{g}$	σ	1.80	2.10
45	10b$_{1g}$	σ	-0.55	-0.39
46	12b$_{3u}$	σ	1.92	2.07
47	14a$_{g}$	σ	2.58	2.36
48	12b$_{2u}$	σ	1.58	1.85
49	15a$_{g}$	σ	1.94	1.84
50	13b$_{2u}$	σ	-0.30	0.39
51	13b$_{3u}$	σ	-0.50	0.03
52	11b$_{1g}$	σ	-2.21	-1.24
53	1b$_{1u}$	π	3.10	3.04
54	16a$_{g}$	σ	1.07	0.72
55	1b$_{3g}$	π	2.78	2.70
56	1b$_{2g}$	π	3.00	2.91
57	14b$_{3u}$	σ	-1.41	-1.64
58	2b$_{1u}$	π	2.60	2.52
59	14b$_{2u}$	σ	-1.59	-2.42
60	17a$_{g}$	σ	-4.10	-3.22
61	12b$_{1g}$	σ	-2.35	-2.30
62	15b$_{3u}$	σ	-3.29	-3.14
63	15b$_{2u}$	σ	-3.79	-3.45
64	16b$_{3u}$	σ	-4.86	-3.31
65	13b$_{1g}$	σ	-5.40	-5.73
66	18a$_{g}$	σ	-5.09	-2.46
67	16b$_{2u}$	σ	-5.08	-2.32
68	14b$_{1g}$	σ	-7.10	-8.58
69	19a$_{g}$	σ	1.16	-0.15
70	17b$_{2u}$	σ	-3.24	-6.49
71	17b$_{3u}$	σ	-3.29	-5.61
72	20a$_{g}$	σ	-3.56	-5.33
73	1a$_{u}$	π	3.45	3.45
74	2b$_{3g}$	π	2.30	2.17
75	2b$_{2g}$	π	0.85	0.66
76	3b$_{1u}$	π	0.54	0.58
77	3b$_{2g}$	π	2.75	2.97
78	4b$_{1u}$	π	0.51	0.58
79	3b$_{3g}$	π	1.49	1.68
80	2a$_{u}$	π	0.69	0.40
81	5b$_{1u}$	π	2.73	2.37
82	4b$_{2g}$	π	-89.97	-73.54

Table S5: The average orbital contributions to the MIRC strength ($\langle I_i \rangle$ in nA/T) of hexadehydro[12]annulene. The calculations were performed at the B3LYP and scLH22t levels. Orbital types and irreducible representations are also given.

№	Orbital	Type	B3LYP	scLH22t
1	1e$'$	core	0.44	0.26
2	1e$'$	core	0.44	0.26
3	1a$'_1$	core	0.20	0.18
4	1a$'_2$	core	1.12	0.97
5	2e$'$	core	0.79	0.81
6	2e$'$	core	0.79	0.81
7	3e$'$	core	0.79	0.72
8	3e$'$	core	0.79	0.72
9	2a$'_1$	core	0.70	0.66
10	2a$'_2$	core	2.20	1.53
11	4e$'$	core	1.46	1.18
12	4e$'$	core	1.46	1.18
13	3a$'_1$	σ	2.89	2.85
14	5e$'$	σ	2.22	2.17
15	5e$'$	σ	2.21	2.18
16	6e$'$	σ	1.49	2.25
17	6e$'$	σ	1.49	2.26
18	4a$'_1$	σ	0.45	1.48
19	3a$'_2$	σ	-0.12	0.36
20	7e$'$	σ	-0.31	0.32
21	7e$'$	σ	-0.31	0.31
22	8e$'$	σ	0.93	1.48
23	8e$'$	σ	0.93	1.48
24	5a$'_1$	σ	1.25	1.86
25	6a$'_1$	σ	-2.70	-3.09
26	9e$'$	σ	-0.38	-1.02
27	9e$'$	σ	-0.38	-1.02
28	4a$'_2$	σ	-2.94	-3.40
29	10e$'$	σ	-5.17	-5.93
30	10e$'$	σ	-5.17	-5.93
31	1a$''_2$	π	3.90	3.89
32	1e$''$	π	3.79	3.78
33	1e$''$	π	3.80	3.77
34	7a$'_1$	σ	-3.06	-3.26
35	2e$''$	π	2.59	2.33
36	2e$''$	π	2.59	2.33
37	11e$'$	σ	-2.26	-2.37
38	11e$'$	σ	-2.27	-2.38
39	2a$''_2$	π	-40.76	-34.94

Table S6: The verage orbital contributions to the MIRC strength ($\langle I_i \rangle$ in nA/T) of planar cyclooctatraene. The calculations were performed at the B3LYP and scLH22t levels. Orbital types and irreducible representations are also given.

№	Orbital	Type	B3LYP	scLH22t
1	$1a_{1g}$	core	0.08	0.04
2	$1e_u$	core	0.05	0.08
3	$1e_u$	core	0.05	0.08
4	$1b_{1g}$	core	0.45	0.37
5	$1b_{2g}$	core	0.38	0.40
6	$2e_u$	core	0.94	0.83
7	$2e_u$	σ	0.93	0.83
8	$1a_{2g}$	σ	1.19	1.12
9	$2a_{1g}$	σ	3.01	3.11
10	$3e_u$	σ	2.72	2.82
11	$3e_u$	σ	2.72	2.82
12	$2b_{1g}$	σ	1.76	1.93
13	$2b_{2g}$	σ	2.28	2.63
14	$4e_u$	σ	1.64	1.75
15	$4e_u$	σ	1.63	1.74
16	$3a_{1g}$	σ	3.11	3.05
17	$4a_{1g}$	σ	0.69	0.72
18	$5e_u$	σ	1.50	1.39
19	$5e_u$	σ	1.49	1.38
20	$2a_{2g}$	σ	-1.62	-1.58
21	$3b_{1g}$	σ	-4.25	-4.60
22	$1a_{2u}$	π	4.10	4.11
23	$6e_u$	σ	-7.38	-7.44
24	$6e_u$	σ	-7.38	-7.45
25	$3b_{2g}$	σ	-6.28	-6.54
26	$1e_g$	π	3.74	3.66
27	$1e_g$	π	3.74	3.65
28	$1b_{2u}$	π	-51.68	-46.47

Table S7: The average orbital contributions to the MIRC strength ($\langle I_i \rangle$ in nA/T) of the bent cyclooctatraene. The calculations were performed at the B3LYP and scLH22t levels. Irreducible representations are also given.

№	Orbital	B3LYP	scLH22t
1	$1a_1$	0.03	0.02
2	1e	0.02	0.03
3	1e	0.02	0.03
4	$1b_2$	0.31	0.23
5	$1b_1$	0.19	0.31

Continued on next page

№	Orbital	B3LYP	scLH22t
6	2e	0.66	0.67
7	2e	0.66	0.67
8	1a$_2$	0.88	0.90
9	2a$_1$	2.93	2.99
10	3e	2.56	2.63
11	3e	2.58	2.60
12	2b$_2$	2.09	2.11
13	2b$_1$	1.86	2.17
14	4e	1.57	1.58
15	4e	1.54	1.61
16	3a$_1$	2.59	2.40
17	5e	1.20	1.09
18	5e	1.22	1.08
19	2a$_2$	-0.44	-0.33
20	4a$_1$	-0.70	-0.59
21	3b$_2$	1.00	0.80
22	6e	-3.44	-3.46
23	6e	-3.41	-3.43
24	3b$_1$	-7.26	-7.40
25	4b$_2$	-0.22	-0.28
26	7e	0.75	0.68
27	7e	0.74	0.67
28	5a$_1$	-12.77	-11.77

Table S8: The average orbital contributions to the MIRC strength ($\langle I_i \rangle$ in nA/T) of cyclobutadiene. The calculations were performed at the B3LYP and scLH22t levels. Orbital types and irreducible representations are also given.

№	Orbital	Type	B3LYP	scLH22t
1	1a$_g$	core	0.02	0.01
2	1b$_{3u}$	core	0.32	0.32
3	1b$_{2u}$	core	0.36	0.46
4	1b$_{1g}$	core	0.61	0.62
5	2a$_g$	σ	3.04	3.16
6	2b$_{3u}$	σ	1.82	2.12
7	2b$_{2u}$	σ	3.04	3.24
8	3a$_g$	σ	1.55	1.40
9	2b$_{1g}$	σ	0.87	1.13
10	3b$_{3u}$	σ	-6.08	-6.79
11	4a$_g$	σ	2.79	2.54
12	1b$_{1u}$	π	3.84	3.85
13	3b$_{2u}$	σ	-12.25	-12.26
14	1b$_{2g}$	π	-19.88	-20.56

Table S9: The average orbital contributions to the MIRC strength ($\langle I_i \rangle$ in nA/T) of the cyclopropenium cation. The calculations were performed at the B3LYP and scLH22t levels. Orbital types, occupation numbers and irreducible representations are also given.

№	Orb.	Occ.	Type	scLH22t def2-TZVP	B3LYP def2-TZVP	B3LYP def2-QZVP	B3LYP cc-pVTZ	B3LYP cc-pVQZ	B3LYP cc-pV5Z	B3LYP cc-pV6Z
1	$1a'_1$	2.0	core	0.01	0.01	0.02	0.03	0.02	0.02	0.02
2	$1e'$	4.0	core	1.29	1.05	1.01	0.76	1.42	1.66	0.05
3	$2a'_1$	2.0	σ	3.02	2.81	2.80	2.80	2.79	2.79	2.79
4	$2e'$	4.0	σ	5.81	5.58	4.66	5.23	5.30	4.91	5.21
5	$3a'_1$	2.0	σ	0.32	0.50	0.51	0.50	0.51	0.51	0.51
6	$1a''_2$	2.0	π	3.92	3.90	3.91	3.90	3.91	3.91	3.91
7	$3e'$	4.0	σ	-4.06	-3.49	-2.47	-2.85	-3.51	-3.36	-2.03

3 The angular dependence of the MIRC strength of the core, valence σ and π orbitals.

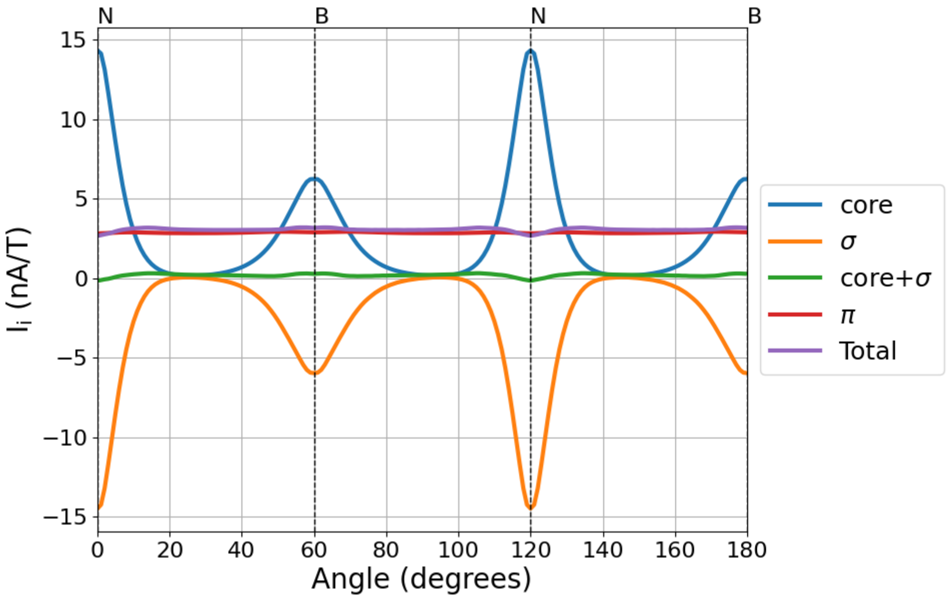

Figure S3: The angular dependence of the MIRC strength of the core, valence σ and π orbitals of borazine. The positions of the nuclei are indicated by vertical dashed lines.

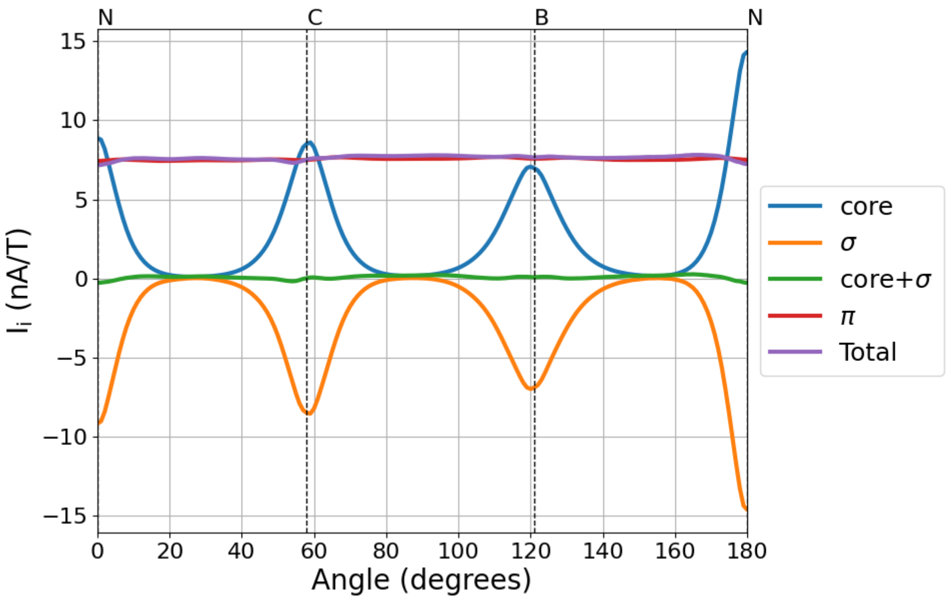

Figure S4: The angular dependence of the MIRC strength of the core, valence σ and π orbitals of $C_2B_2N_2H_6$. The positions of the nuclei are indicated by vertical dashed lines.

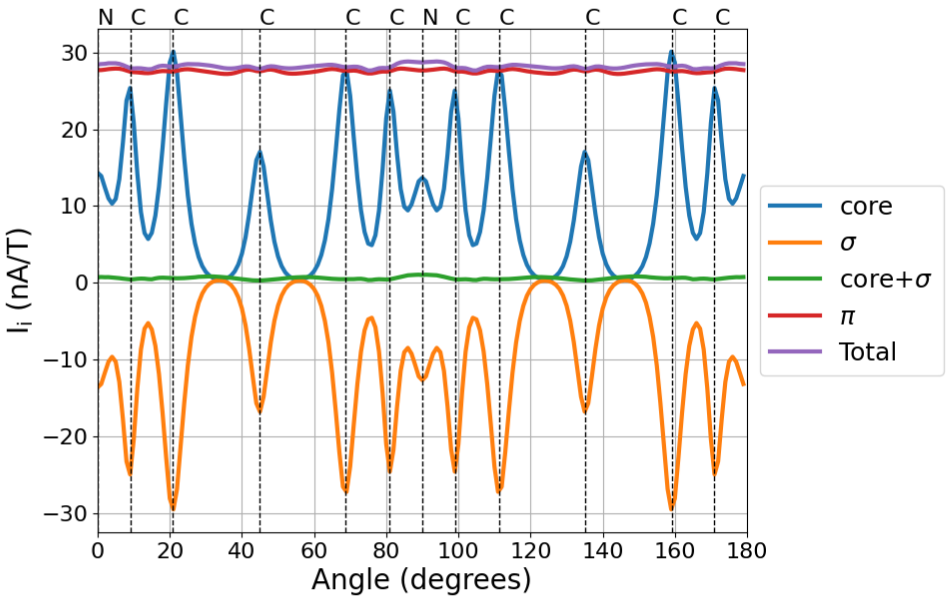

Figure S5: The angular dependence of the MIRC strength of the core, valence σ and π orbitals of porphin. The positions of the nuclei are indicated by vertical dashed lines.

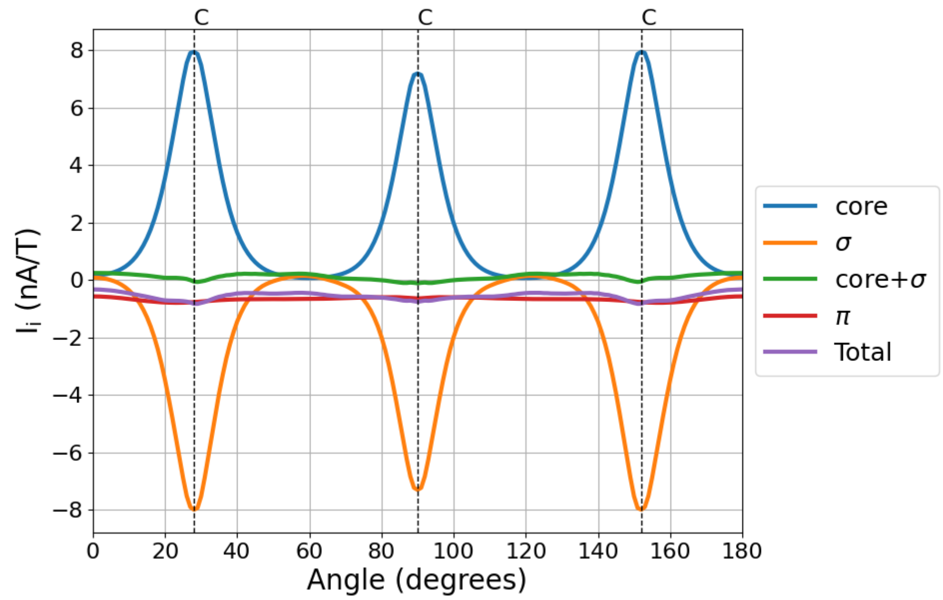

Figure S6: The angular dependence of the MIRC strength of the core, valence σ and π orbitals of 1,4-cyclohexadiene. The positions of the nuclei are indicated by vertical dashed lines.

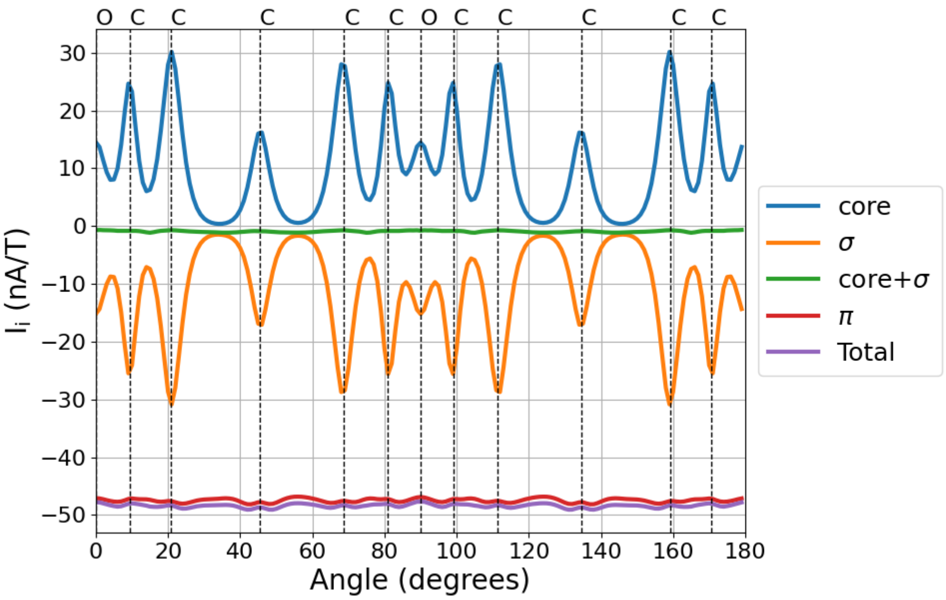

Figure S7: The angular dependence of the MIRC strength of the core, valence σ and π orbitals of tetraoxa-isophlorin. The positions of the nuclei are indicated by vertical dashed lines.

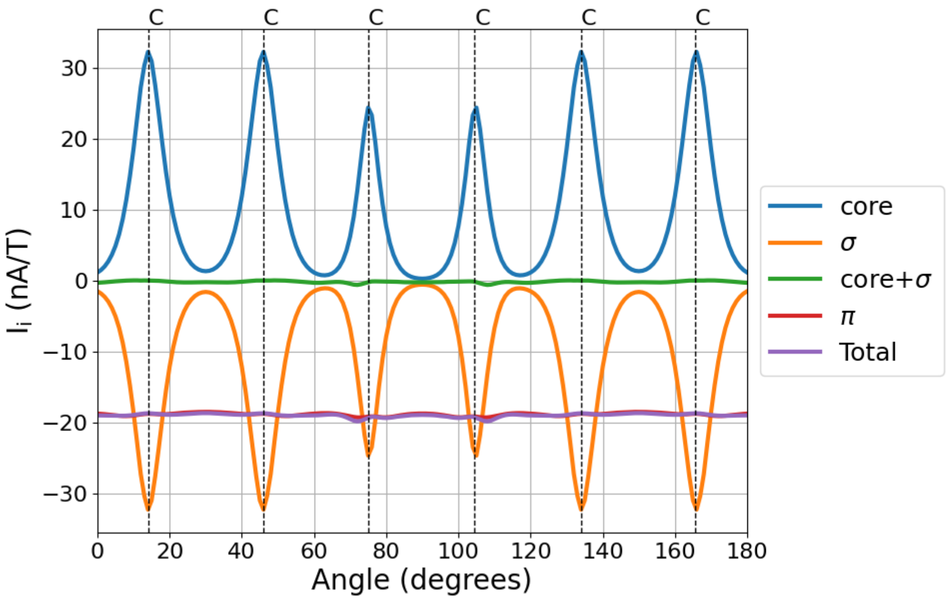

Figure S8: The angular dependence of the MIRC strength of the core, valence σ and π orbitals of hexadehydro[12]annulene. The positions of the nuclei are indicated by vertical dashed lines.

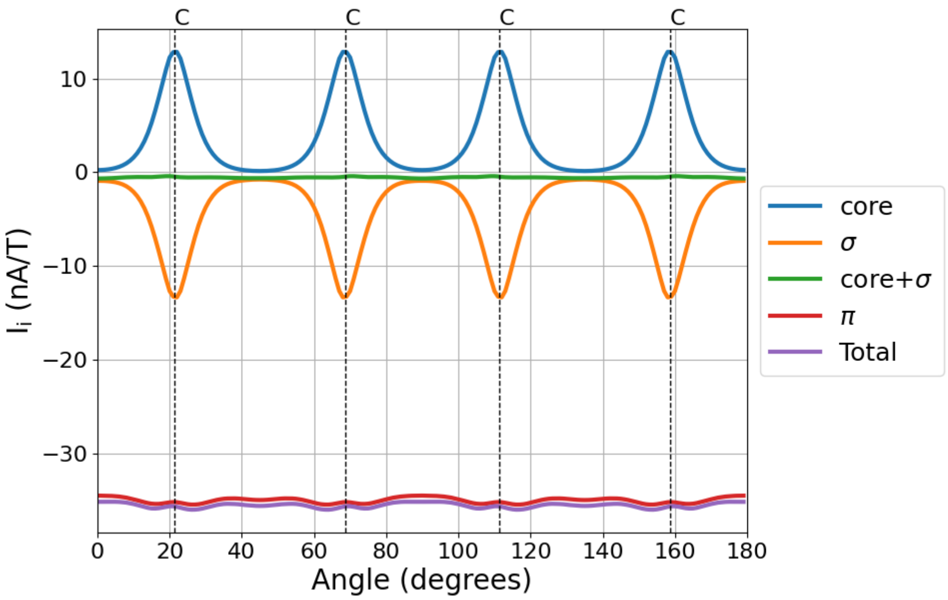

Figure S9: The angular dependence of the MIRC strength of the core, valence σ and π orbitals of planar cyclooctatraene. The positions of the nuclei are indicated by vertical dashed lines.

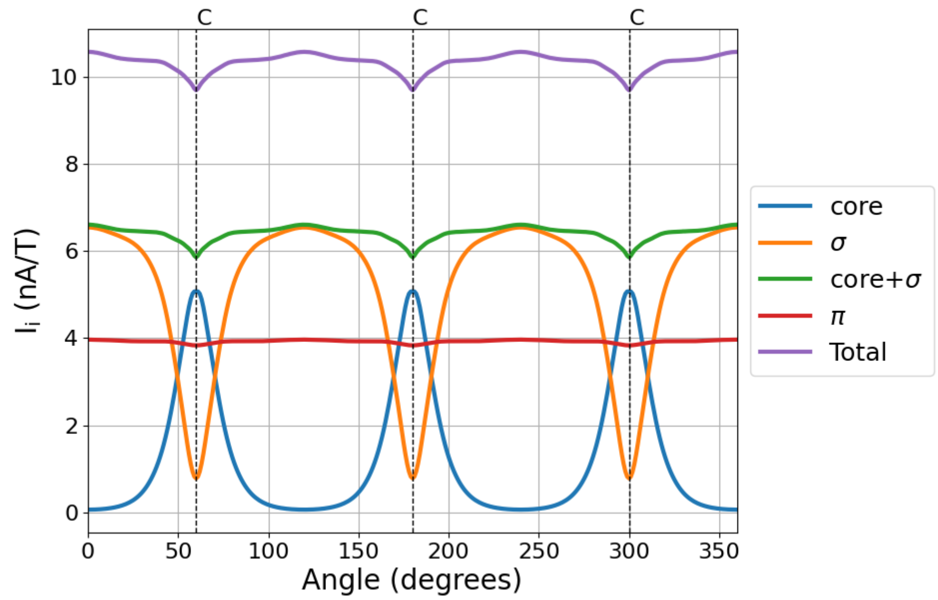

Figure S10: The angular dependence of the MIRC strength of the core, valence σ and π orbitals of the cyclopropenium cation calculated using the def2-TZVP basis set. The positions of the nuclei are indicated by vertical dashed lines.

4 The angular dependence of the MIRC strength of all orbitals of each irreducible representation

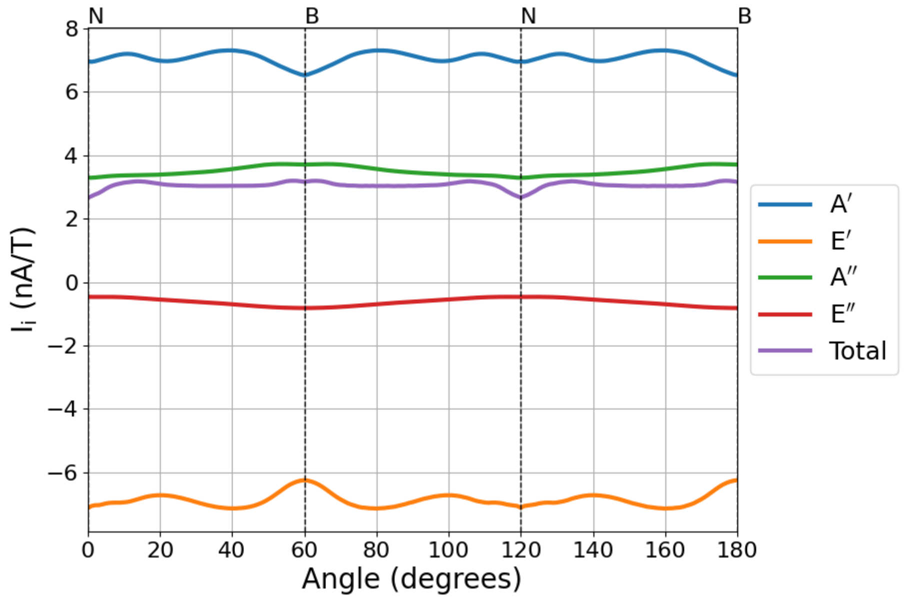

Figure S11: The angular dependence of the MIRC strength of borazine for all orbitals of each irreducible representation of the C_{3h} point group.

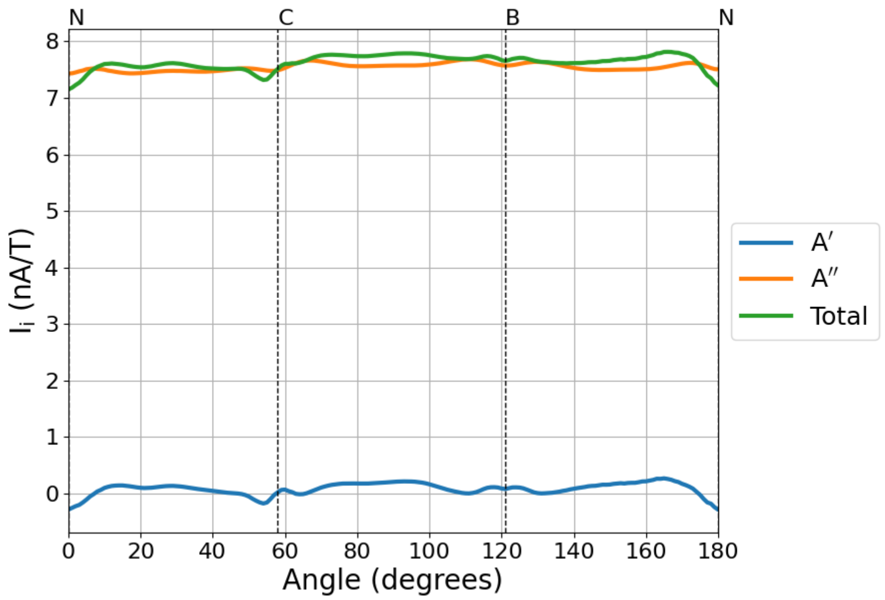

Figure S12: The angular dependence of the MIRC strength of $C_2B_2N_2H_6$ for all orbitals of each irreducible representation of the C_s point group.

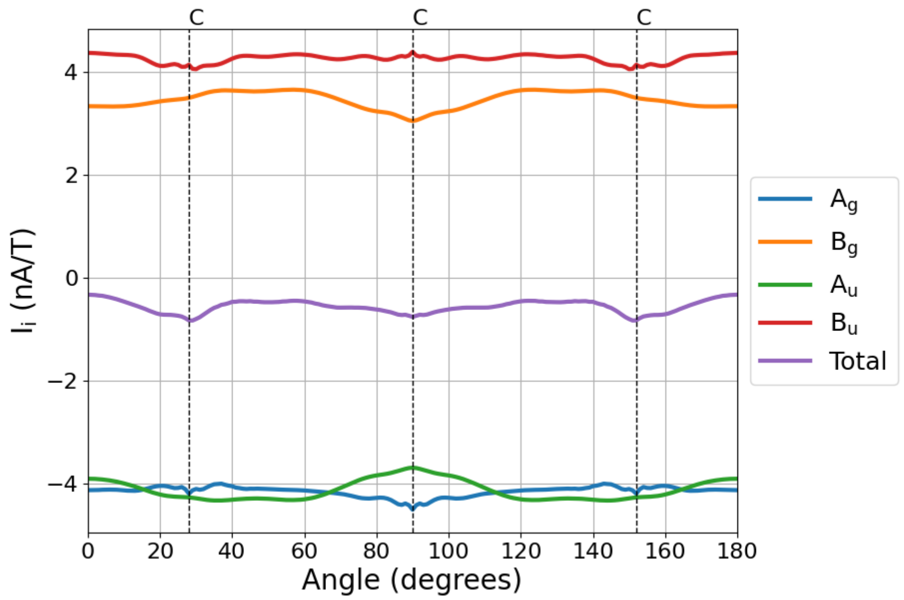

Figure S13: The angular dependence of the MIRC strength of 1,4-cyclohexadiene for all orbitals of each irreducible representation of the C_{2h} point group.

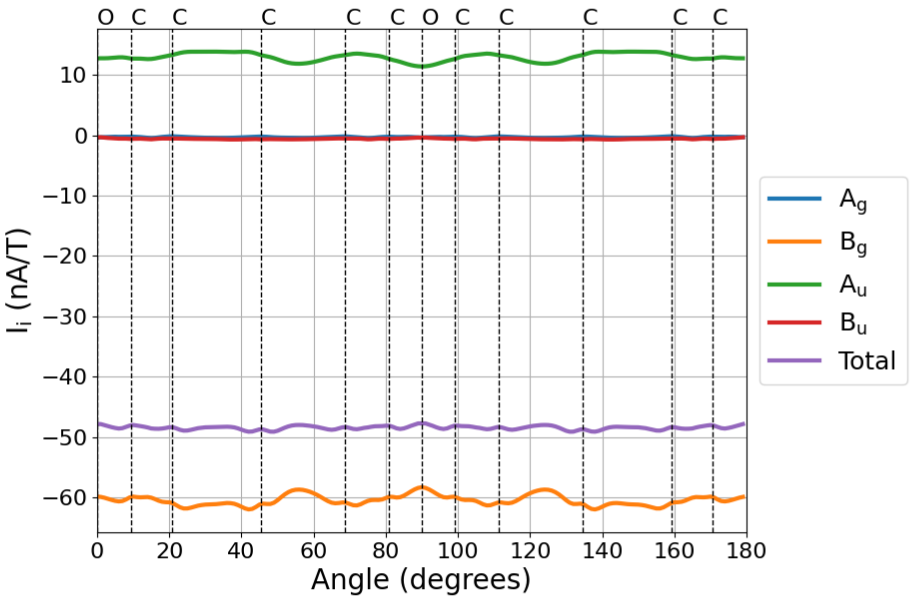

Figure S14: The angular dependence of the MIRC strength of tetraoxa-isophlorin for all orbitals of each irreducible representation of the C_{2h} point group.

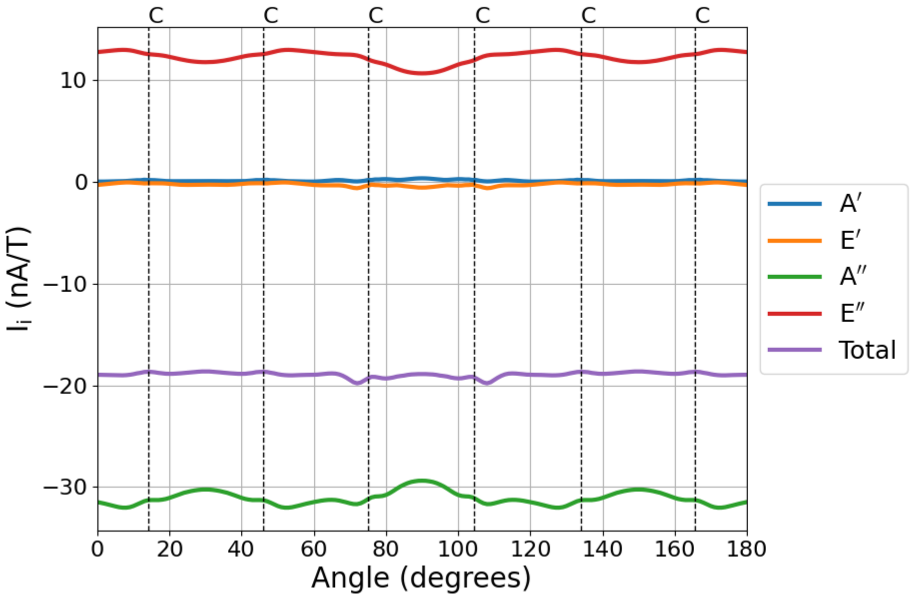

Figure S15: The angular dependence of the MIRC strength of hexadehydro[12]annulene for all orbitals of each irreducible representation of the C_{3h} point group.

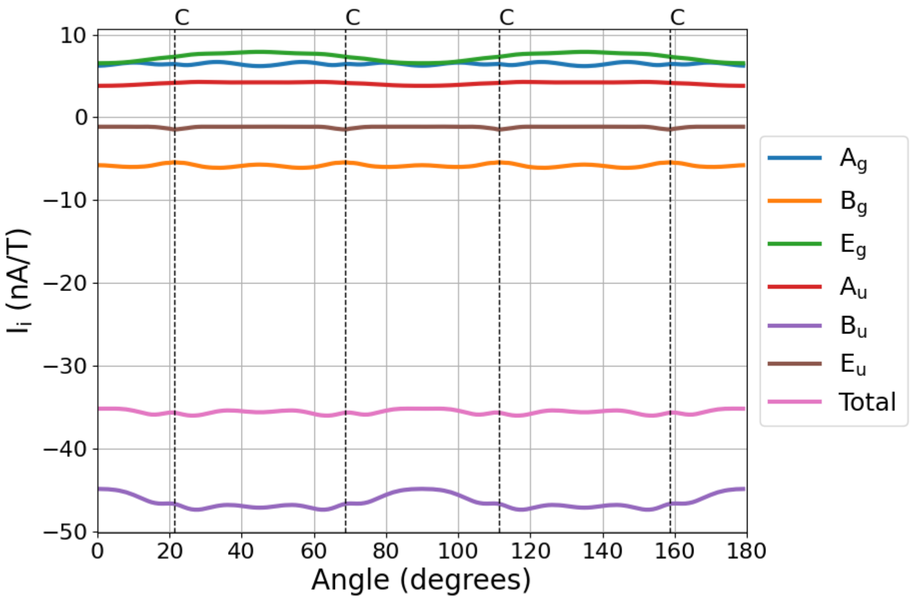

Figure S16: The angular dependence of the MIRC strength of planar cyclooctatraene for all orbitals of each irreducible representation of the C_{4h} point group.

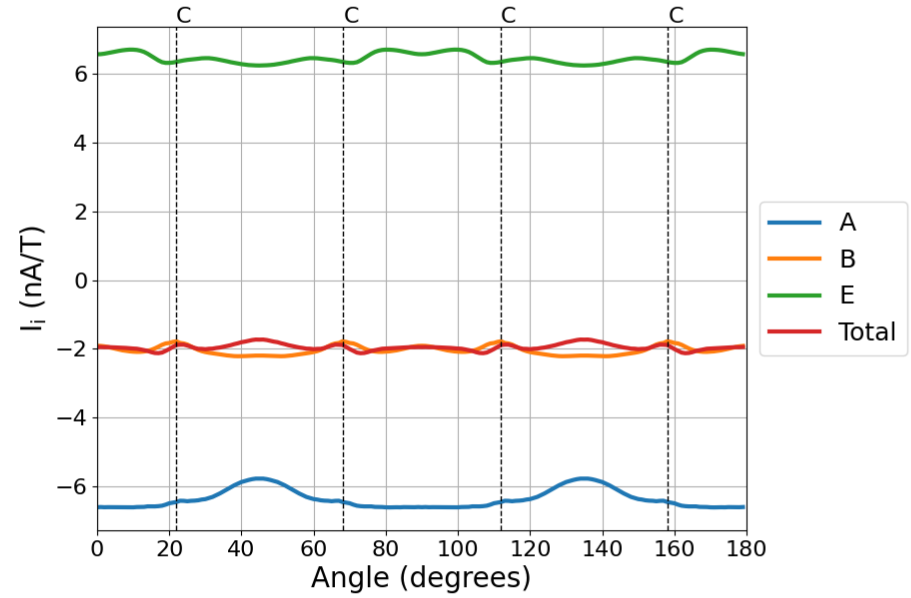

Figure S17: The angular dependence of the MIRC strength of bent cyclooctatraene for all orbitals of each irreducible representation of the S_4 point group.

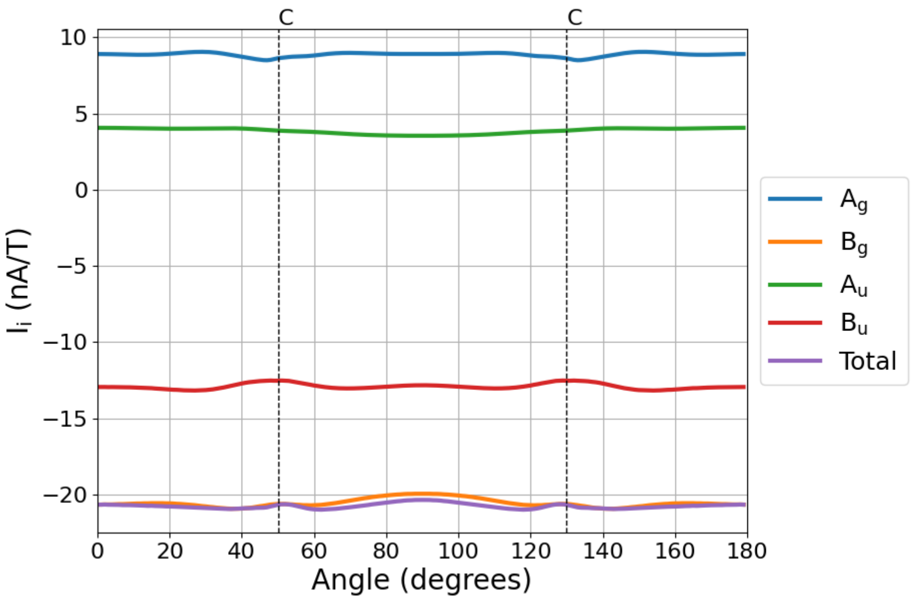

Figure S18: The angular dependence of the MIRC strength of cyclobutadiene for all orbitals of each irreducible representation of the C_{2h} point group.

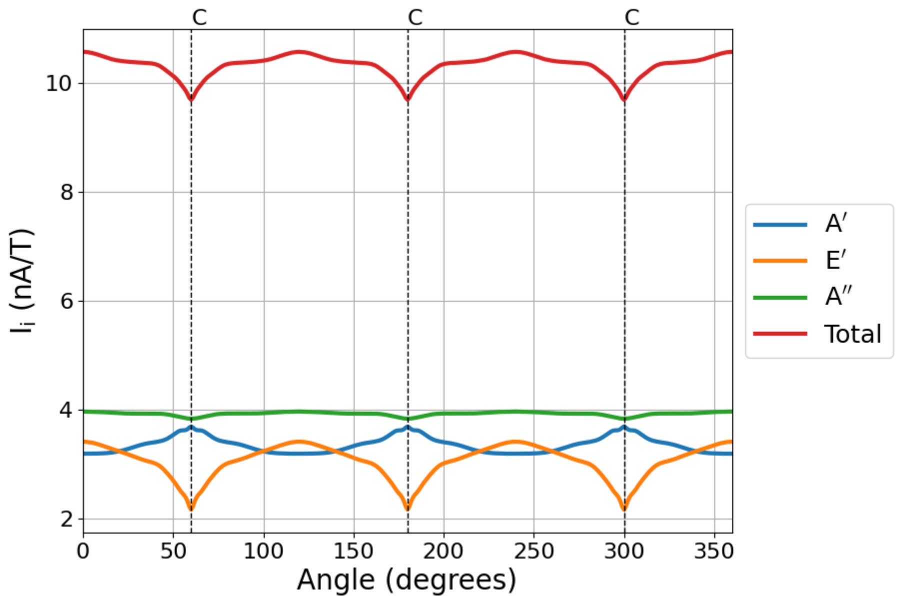

Figure S19: The angular dependence of the MIRC strength of the cyclopropenium cation calculated using the def2-TZVP basis set for all orbitals of each irreducible representation of the C_{3h} point group.

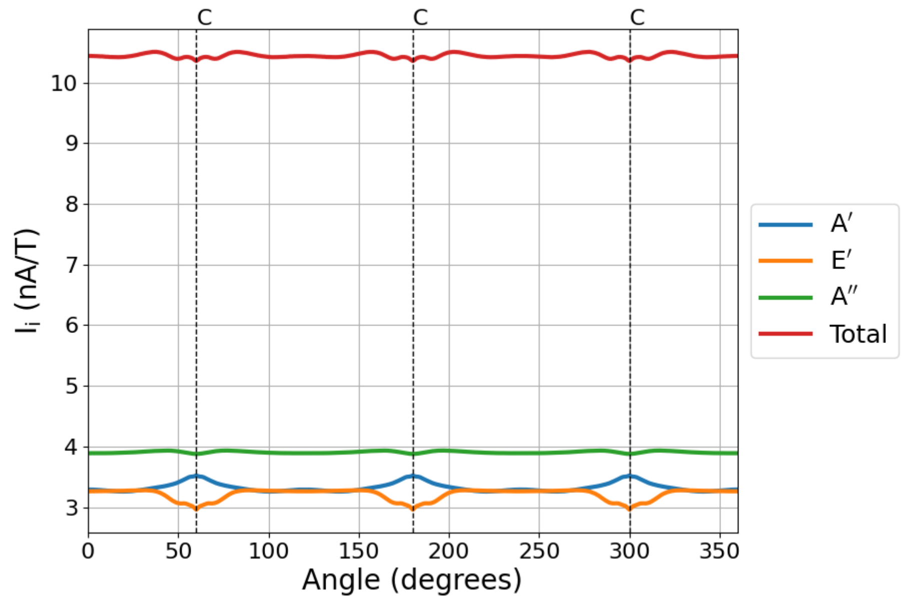

Figure S20: The angular dependence of the MIRC strength of the cyclopropenium cation calculated using the def2-QZVP basis set for all orbitals of each irreducible representation of the C_{3h} point group.

5 Optimized geometries of the studied molecules.

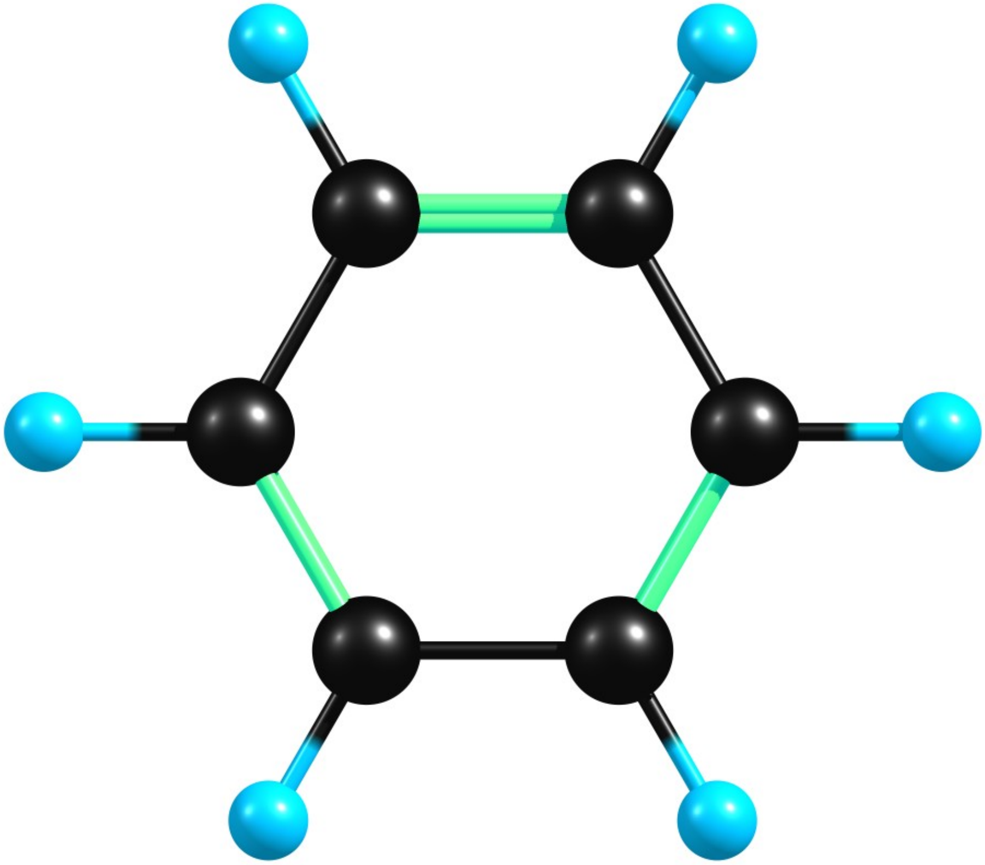

Figure S21: Optimized geometry of the benzene.

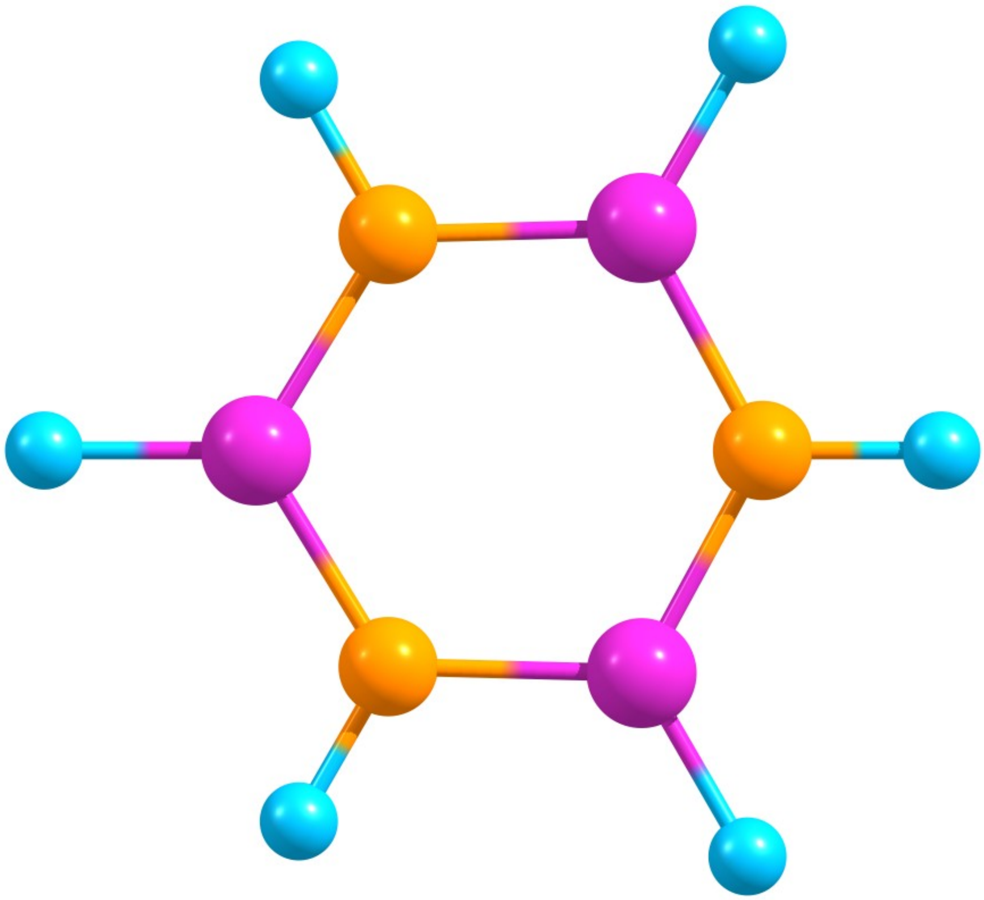

Figure S22: Optimized geometry of the borazine.

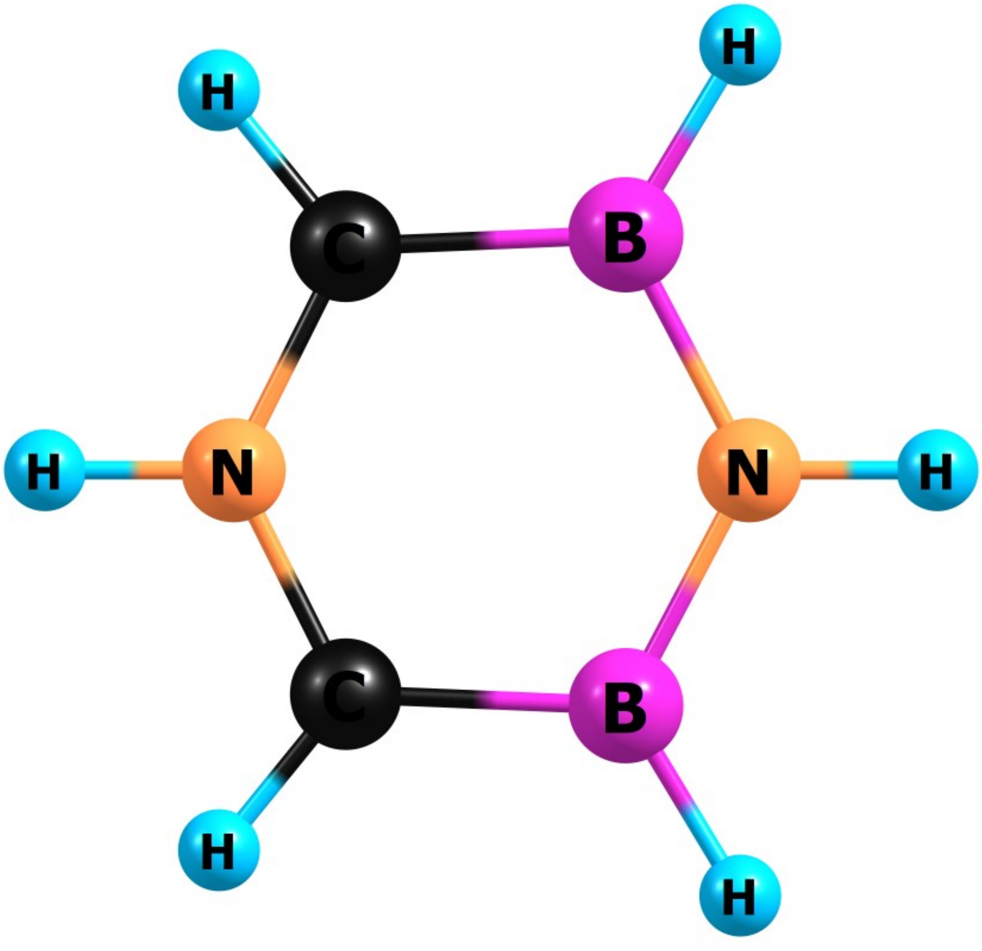

Figure S23: Optimized geometry of the $C_2B_2N_2$.

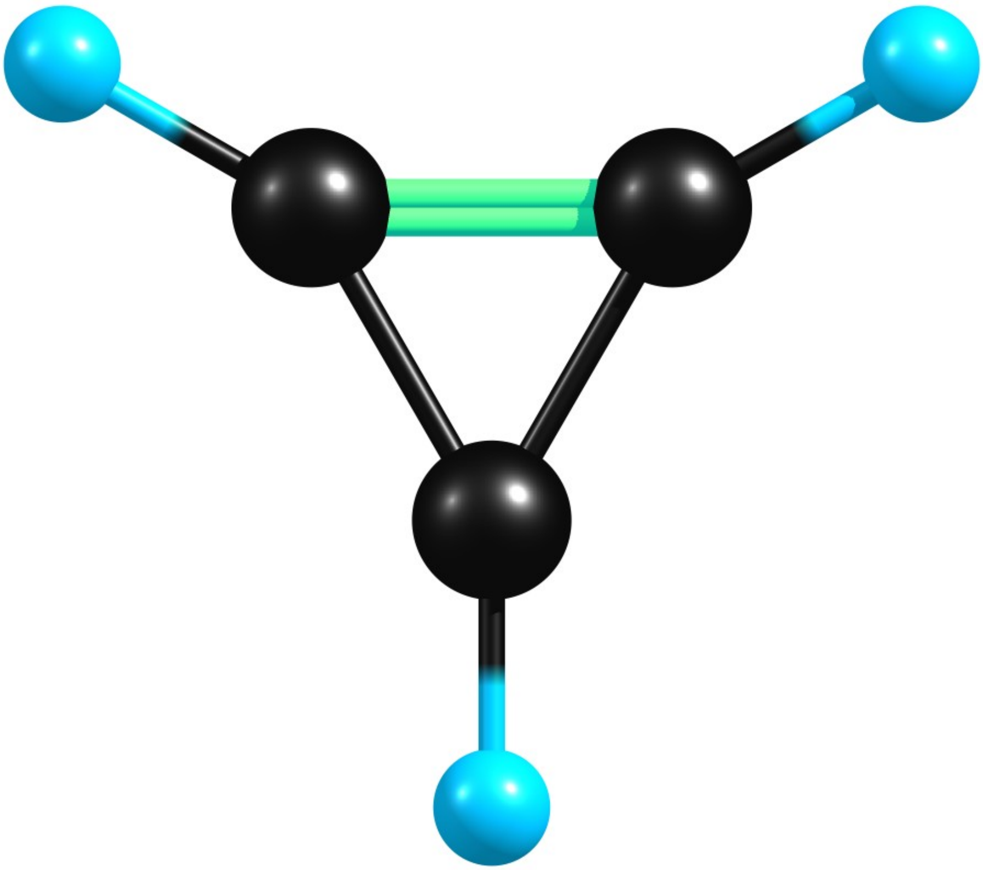

Figure S24: Optimized geometry of the $C_3H_3{}^+$.

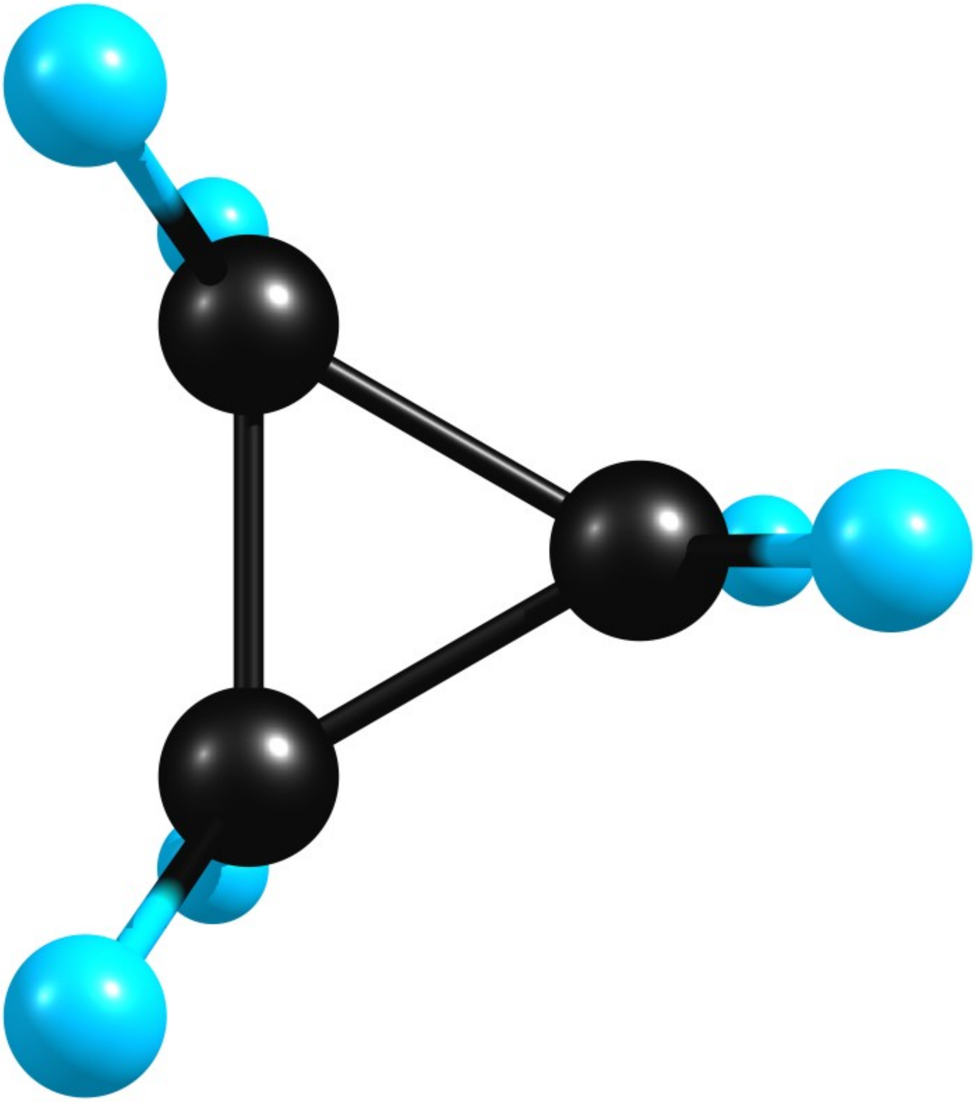

Figure S25: Optimized geometry of the cyclopropane.

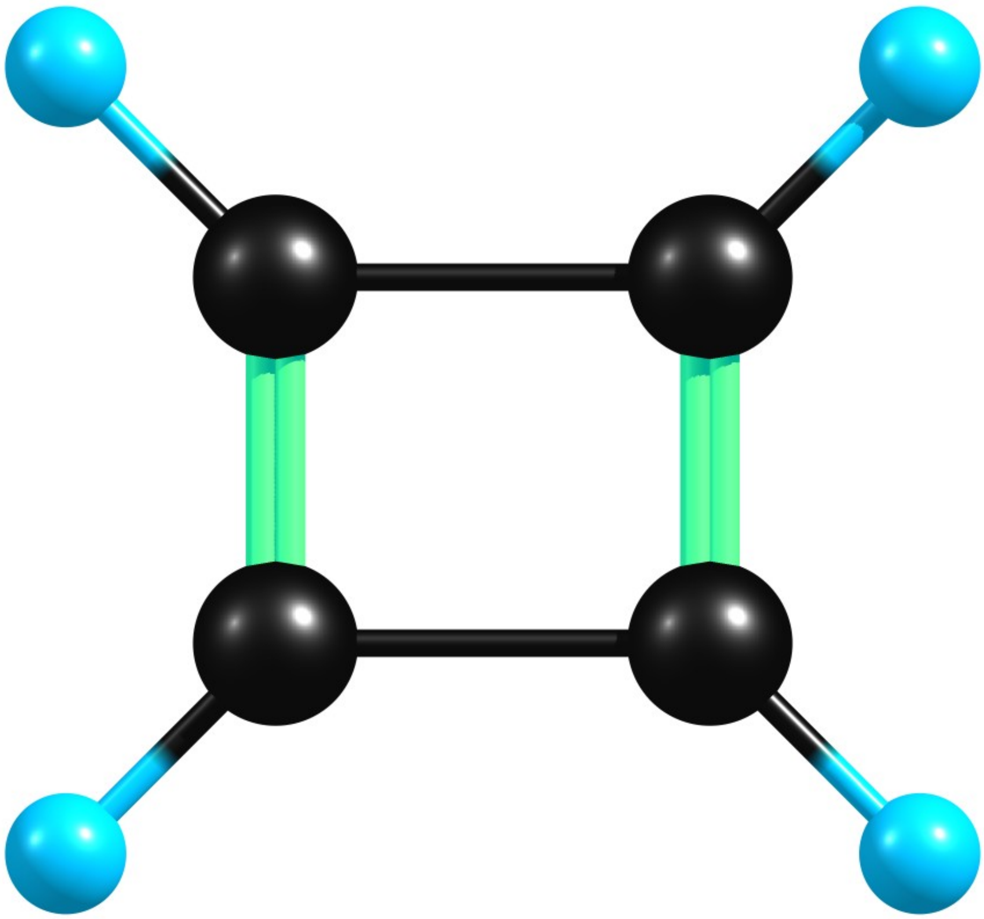

Figure S26: Optimized geometry of the C_4H_4.

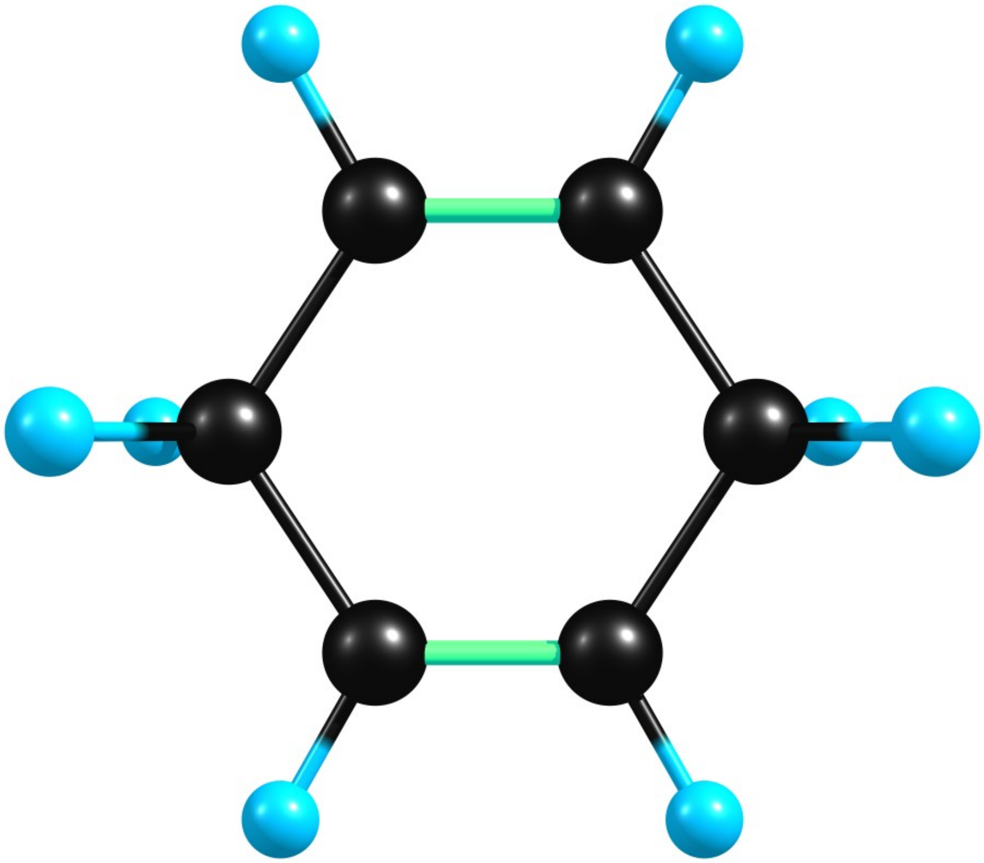

Figure S27: Optimized geometry of the 1,4-cyclohexadiene.

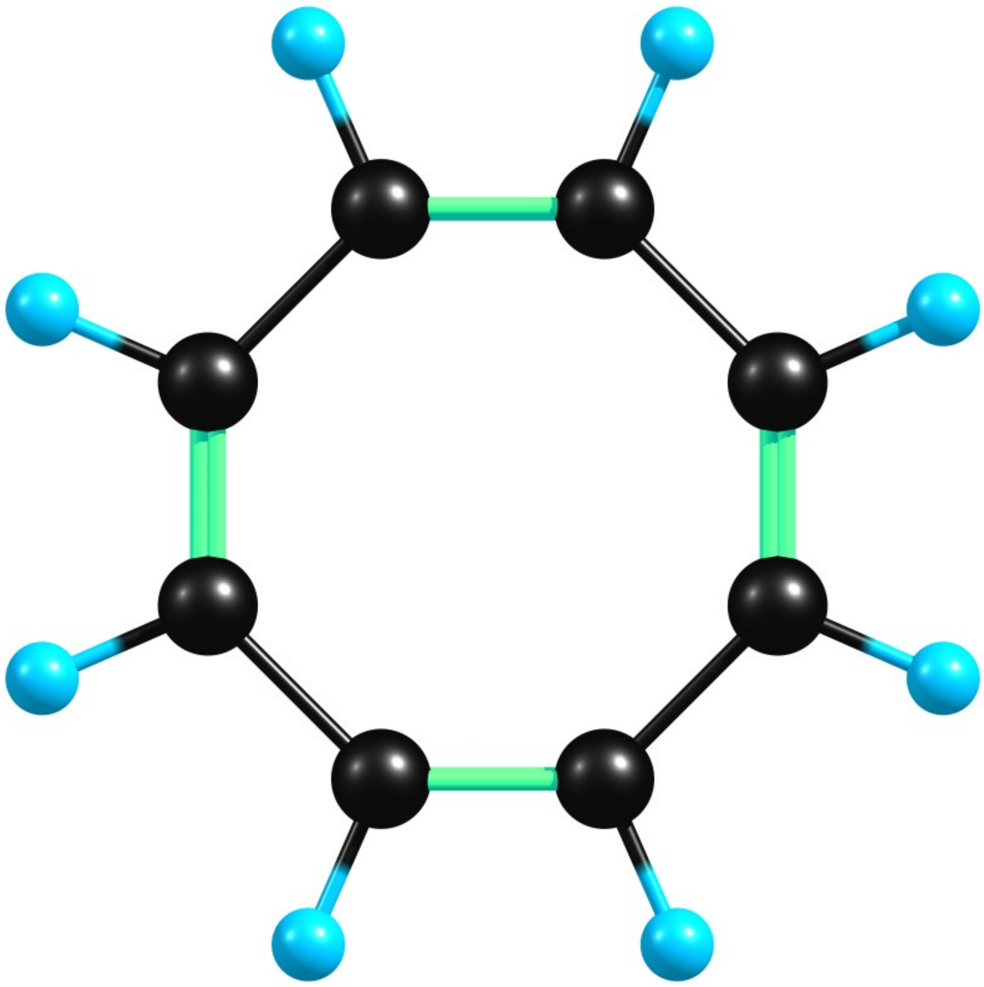

Figure S28: Optimized geometry of the planar COT.

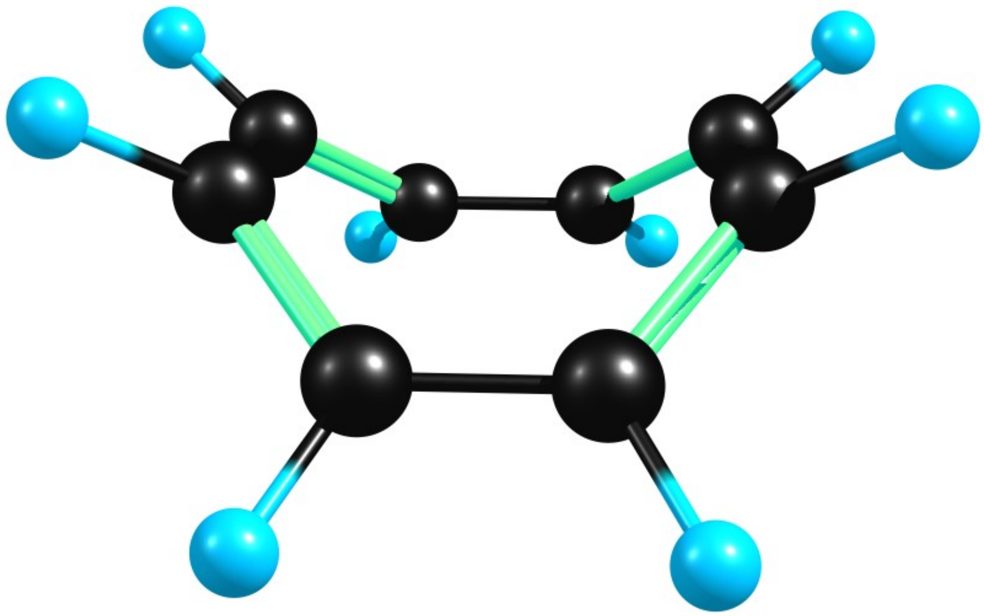

Figure S29: Optimized geometry of the bent COT.

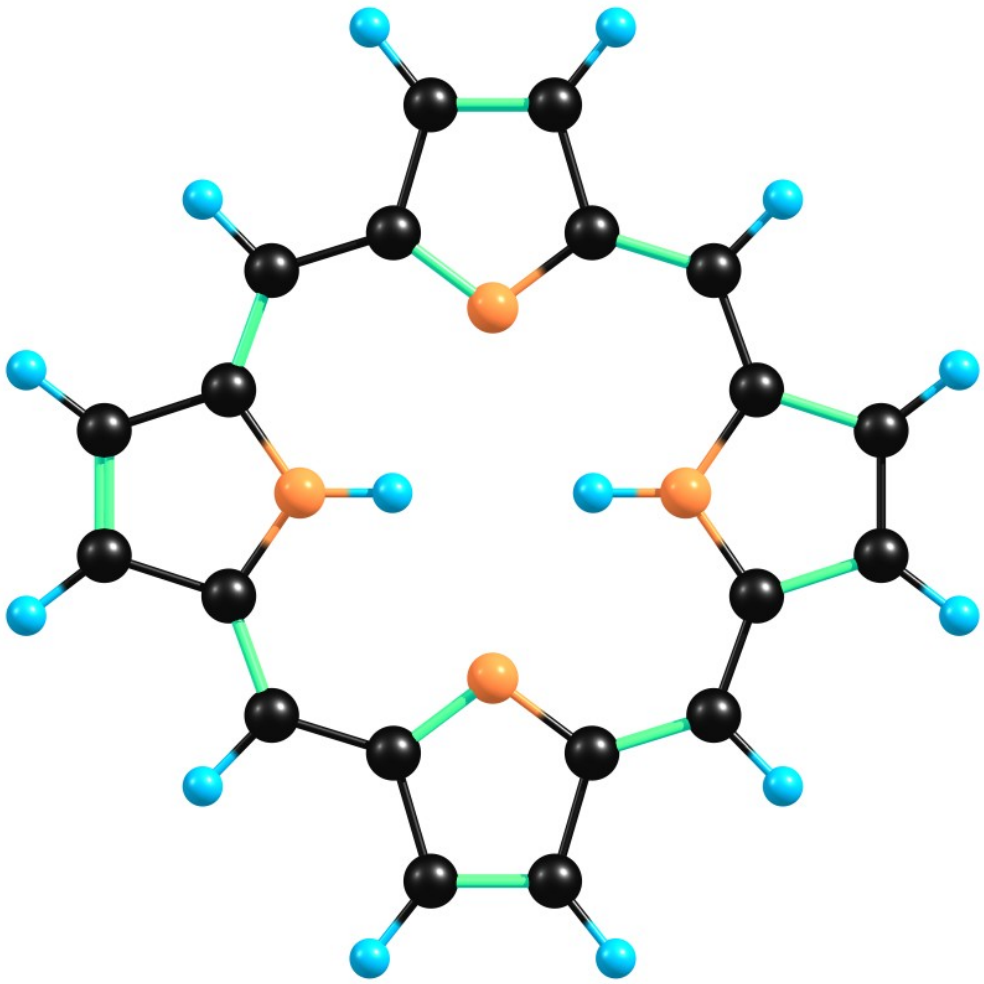

Figure S30: Optimized geometry of the porphin.

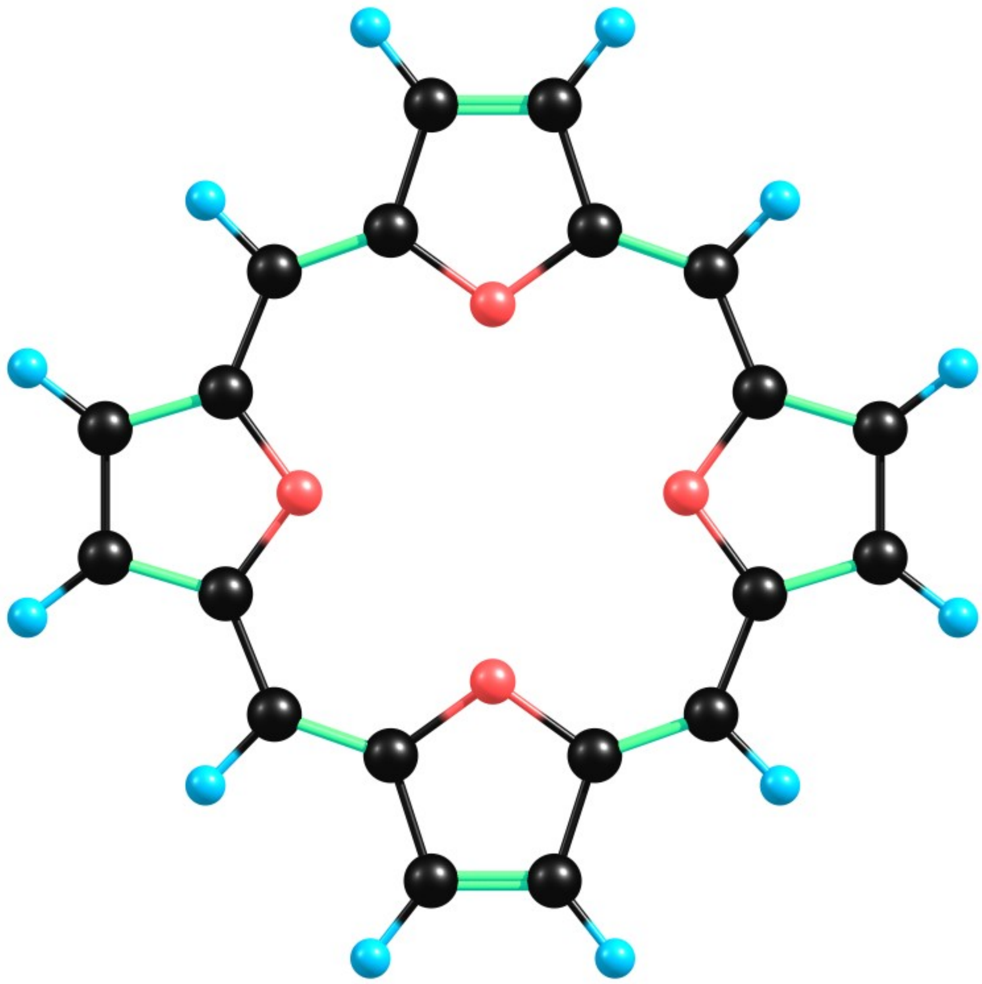

Figure S31: Optimized geometry of the isophlorin.

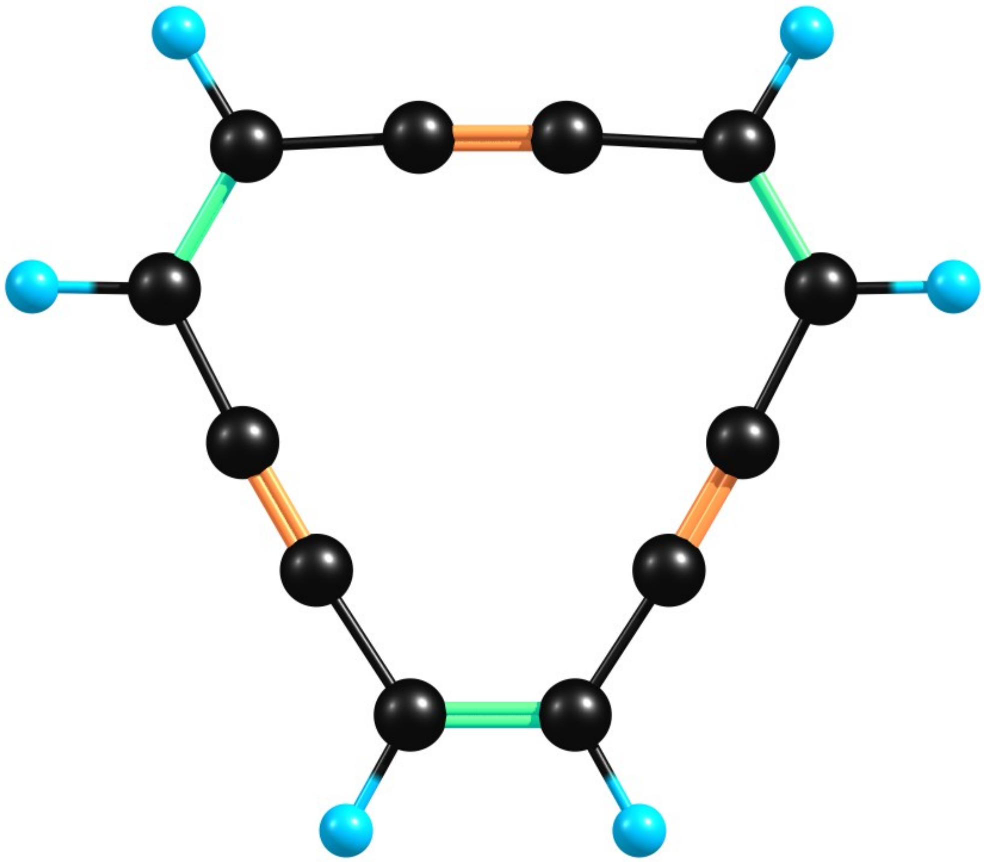

Figure S32: Optimized geometry of the hexadehydroannulene.
